# M1 muscarinic allosteric modulators slow prion neurodegeneration and restore memory loss

**DOI:** 10.1172/JCI87526

**Published:** 2016-12-19

**Authors:** Sophie J. Bradley, Julie-Myrtille Bourgognon, Helen E. Sanger, Nicholas Verity, Adrian J. Mogg, David J. White, Adrian J. Butcher, Julie A. Moreno, Colin Molloy, Timothy Macedo-Hatch, Jennifer M. Edwards, Jurgen Wess, Robert Pawlak, David J. Read, Patrick M. Sexton, Lisa M. Broad, Joern R. Steinert, Giovanna R. Mallucci, Arthur Christopoulos, Christian C. Felder, Andrew B. Tobin

**Affiliations:** 1 The Centre for Translational Pharmacology, Institute of Molecular, Cell and Systems Biology, College of Medical, Veterinary and Life Sciences, University of Glasgow, Glasgow, United Kingdom.; 2 MRC Toxicology Unit, University of Leicester, Leicester, United Kingdom.; 3 Eli Lilly and Co., Neuroscience, Windlesham, Surrey, United Kingdom.; 4 Central Research Facility, University of Leicester, Leicester, United Kingdom.; 5 Molecular Signaling Section, Laboratory of Bioorganic Chemistry, National Institute of Diabetes and Digestive and Kidney Diseases, Bethesda, Maryland, USA.; 6 Institute of Biomedical and Clinical Science, University of Exeter Medical School, University of Exeter, Exeter, United Kingdom.; 7 Drug Discovery Biology, Monash Institute of Pharmaceutical Sciences and Department of Pharmacology, Monash University, Parkville, Victoria, Australia.; 8 Department of Clinical Neurosciences, University of Cambridge, Cambridge, United Kingdom.; 9 Eli Lilly and Co., Neuroscience, Lilly Corporate Center, Indianapolis, Indiana, USA.

## Abstract

The current frontline symptomatic treatment for Alzheimer’s disease (AD) is whole-body upregulation of cholinergic transmission via inhibition of acetylcholinesterase. This approach leads to profound dose-related adverse effects. An alternative strategy is to selectively target muscarinic acetylcholine receptors, particularly the M1 muscarinic acetylcholine receptor (M1 mAChR), which was previously shown to have procognitive activity. However, developing M1 mAChR–selective orthosteric ligands has proven challenging. Here, we have shown that mouse prion disease shows many of the hallmarks of human AD, including progressive terminal neurodegeneration and memory deficits due to a disruption of hippocampal cholinergic innervation. The fact that we also show that muscarinic signaling is maintained in both AD and mouse prion disease points to the latter as an excellent model for testing the efficacy of muscarinic pharmacological entities. The memory deficits we observed in mouse prion disease were completely restored by treatment with benzyl quinolone carboxylic acid (BQCA) and benzoquinazoline-12 (BQZ-12), two highly selective positive allosteric modulators (PAMs) of M1 mAChRs. Furthermore, prolonged exposure to BQCA markedly extended the lifespan of diseased mice. Thus, enhancing hippocampal muscarinic signaling using M1 mAChR PAMs restored memory loss and slowed the progression of mouse prion disease, indicating that this ligand type may have clinical benefit in diseases showing defective cholinergic transmission, such as AD.

## Introduction

Due to an aging population, it is predicted that by 2040, neurodegenerative diseases, such as Alzheimer’s disease (AD), will be the second most common cause of morbidity in the developed world, after cancer (1). The current frontline symptomatic treatment for AD are acetylcholinesterase (AChE) inhibitors (2). These drugs act to increase cholinergic transmission, which is reduced in AD due to a loss of cholinergic innervation to key brain regions (3, 4). Despite some clinical benefit, the nonselective mode of action of AChE inhibitors results in significant dose-related adverse effects that can limit clinical usage (2).

To circumvent these adverse effects, more selective approaches to the improvement of cholinergic transmission specifically associated with memory have been pursued. In particular, activation of the M1 muscarinic acetylcholine receptor (M1 mAChR) has procognitive effects (5–8), making this muscarinic receptor subtype an attractive therapeutic target for AD (7). However, due to the highly conserved nature of the muscarinic receptor orthosteric ligand-binding site (9, 10), the identification of M1 mAChR–selective ligands has been challenging (11). This lack of receptor subtype selectivity has resulted in the failure of numerous M1 mAChR drug discovery programs, exemplified by the mAChR ligand xanomeline — a drug that relieved some of the behavioral and cognitive deficits in AD patients (12, 13), but that failed in clinical trials due to side effects possibly due to the fact that, in addition to M1 mAChRs, xanomeline has significant action on other muscarinic receptor subtypes, including cardiac M_2_ mAChRs and peripheral M_3_ mAChRs (14, 15).

An alternative strategy would be to target the M1 mAChR with positive allosteric modulators (PAMs) that bind to spatially distinct allosteric sites located within nonconserved receptor regions and thereby display exquisite receptor-subtype selectivity (16, 17). Although muscarinic receptor PAMs have been shown to have an impact on learning and memory (18–21), these ligands have not been tested in models of progressive neurodegeneration that ultimately results in end-stage disease. Testing the action of M1 mAChR PAMs in the context of disease where cholinergic transmission is compromised, as it is in AD, is important given the fact that PAMs can act by enhancing the action of the endogenous ligand acetylcholine, thereby maintaining the spatiotemporal signaling of the endogenous ligand (17, 22), and/or by directly activating the receptor as so-called “PAM agonists.” Establishing which of these primary mechanisms operates to affect memory and learning in a disease setting of end-stage neurodegeneration is fundamental to understanding the clinical potential of muscarinic receptor PAMs.

The challenge lies in the fact that none of the current mouse AD models feature continued neuronal loss that ultimately results in terminal disease (23); thus, the impact and mechanism of action of muscarinic ligands in relieving symptoms of neurodegeneration or modification of disease progression in the context of progressive neurodegenerative disease have not yet been tested (24).

In contrast to mouse AD models, mouse prion disease, which is characterized by an accumulation of misfolded prion protein aggregates (3, 15, 25), results in progressive neuronal loss and eventual terminal disease (24, 26, 27). Here, we show that mouse prion disease shares a number of key features with AD that include a loss of hippocampal cholinergic innervation and a cholinergic deficit that results in defective learning and memory while maintaining postsynaptic muscarinic receptors and signaling. These findings prompted us to test the activity of muscarinic ligands in prion disease, where we determined that the prion-mediated deficit in learning and memory was completely restored by the muscarinic receptor agonist xanomeline (28, 29) as well as by the highly selective M1 mAChR PAMs benzyl quinolone carboxylic acid (BQCA) (30–32) and benzoquinazoline-12 (BQZ-12) (33). Furthermore, since the mechanism of action of the M1 mAChR PAMs resulted in reduced adverse effects, we were able to test whether prolonged treatment with BQCA could slow disease progression. We found that daily dosing with BQCA significantly (*P* < 0.001) increased the survival of prion-diseased mice, indicating that M1 mAChR–selective PAMs have the potential to achieve clinical efficacy in alleviating cognitive deficits in neurodegenerative disease as well as to slow disease progression.

## Results

### M1 mAChR in hippocampal learning and memory.

**Figure 1:**
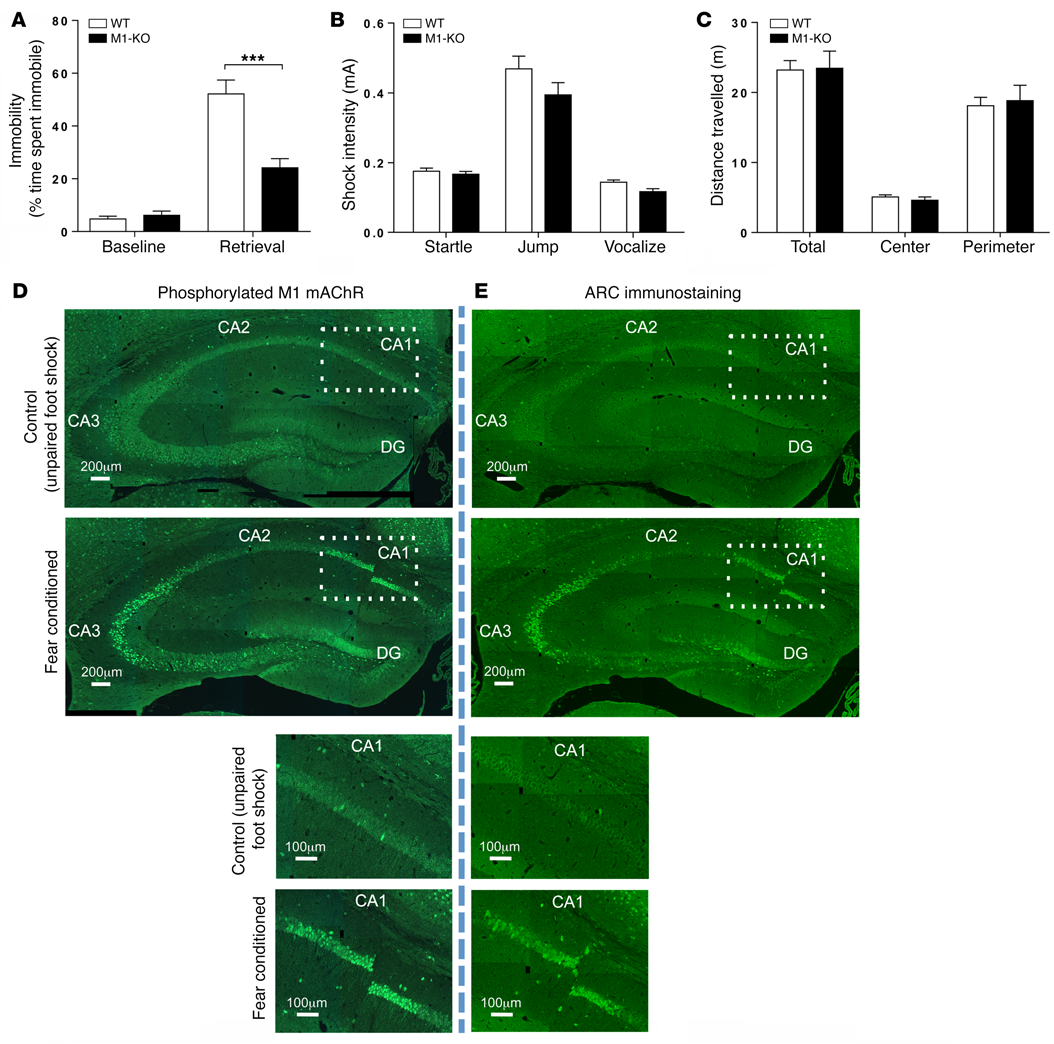
M1 mAChRs play an important role in hippocampal-dependent learning and memory. (**A**) Fear-conditioning response of WT and M1-KO mice. Statistical analysis by 2-way ANOVA with Sidak’s multiple comparison test. ***P < 0.001. (**B**) Pain thresholds for WT and M1-KO mice. Statistical analysis by Student’s *t* test. (**C**) Locomotion of WT and M1-KO mice was determined by total distance traveled during an open field test. Data were analyzed using 2-way ANOVA with Sidak’s multiple comparisons. All WT and M1-KO behavioral data are shown as mean ± SEM of *n* = 8 mice. (**D**) An antibody-based biosensor for M1 mAChR activation (phosphorylation of the M1 mAChR on S228 in the third intracellular loop) was used to assess M1 mAChR activity in the hippocampus. Following fear-conditioning training, phosphorylation at S228 of the M1 mAChR was increased in the CA1 and CA3 regions and dentate gyrus of the hippocampus relative to control mice that received a 2-second unpaired foot shock. Magnification of the CA1 region (indicated by the rectangle) is shown in lower panels. (**E**) Fear-conditioning training increased neuronal activity, as assessed by an increase in ARC immunostaining, in the same regions of the hippocampus as those observed for activated M1 mAChR. **D** and **E** are composited images. Magnification of the CA1 region (indicated by the rectangle) is shown in lower panels (see **D** and **E**). Scale bars: 200 μm (upper panels); 100 μm (lower panels).

We used a series of mAChR knockout mice, in combination with radioligand binding, using the nonselective muscarinic receptor antagonist [^3^H]-*N*-methyl scopolamine ([^3^H]-NMS]) to establish that the predominant mAChR subtype expressed in the hippocampus of mice is the M1 mAChR (Supplemental Figure 1; supplemental material available online with this article; https://doi.org/10.1172/JCI87526DS1). In contextual fear conditioning, a behavioral test of hippocampal-dependent learning and memory (34), M1 mAChR knockout (M1-KO) mice demonstrated a significant deficit (Figure 1A), whereas pain threshold and locomotor activity were not altered (Figure 1, B and C).

To further probe the involvement of the M1 mAChR in hippocampal-dependent learning and memory, we investigated the activation status of the M1 mAChR following fear-conditioning training using an antibody-based biosensor of receptor activation. This biosensor was developed from mass spectrometry–based (MS-based) phospho-proteomics on the M1 mAChR that identified phosphorylation at serine 228 (S228) in the third intracellular loop of the receptor as highly sensitive to agonist stimulation (35) (Supplemental Figure 2A). Using this antibody biosensor for M1 mAChR activation, we determined that M1 mAChR activity was increased in the dentate gyrus and CA3 and CA1 regions of the hippocampus following fear conditioning (Figure 1D), regions that also showed increased neuronal activity, as determined by the increased expression of markers for neuronal activity-related cytoskeleton-associated protein (ARC) (Figure 1E) and cFOS (Supplemental Figure 2B). These behavioral and biochemical data indicate an important role for M1 mAChR activation in hippocampal-dependent learning and memory.

### Cholinergic innervation and muscarinic receptor signaling in prion disease.

**Figure 2:**
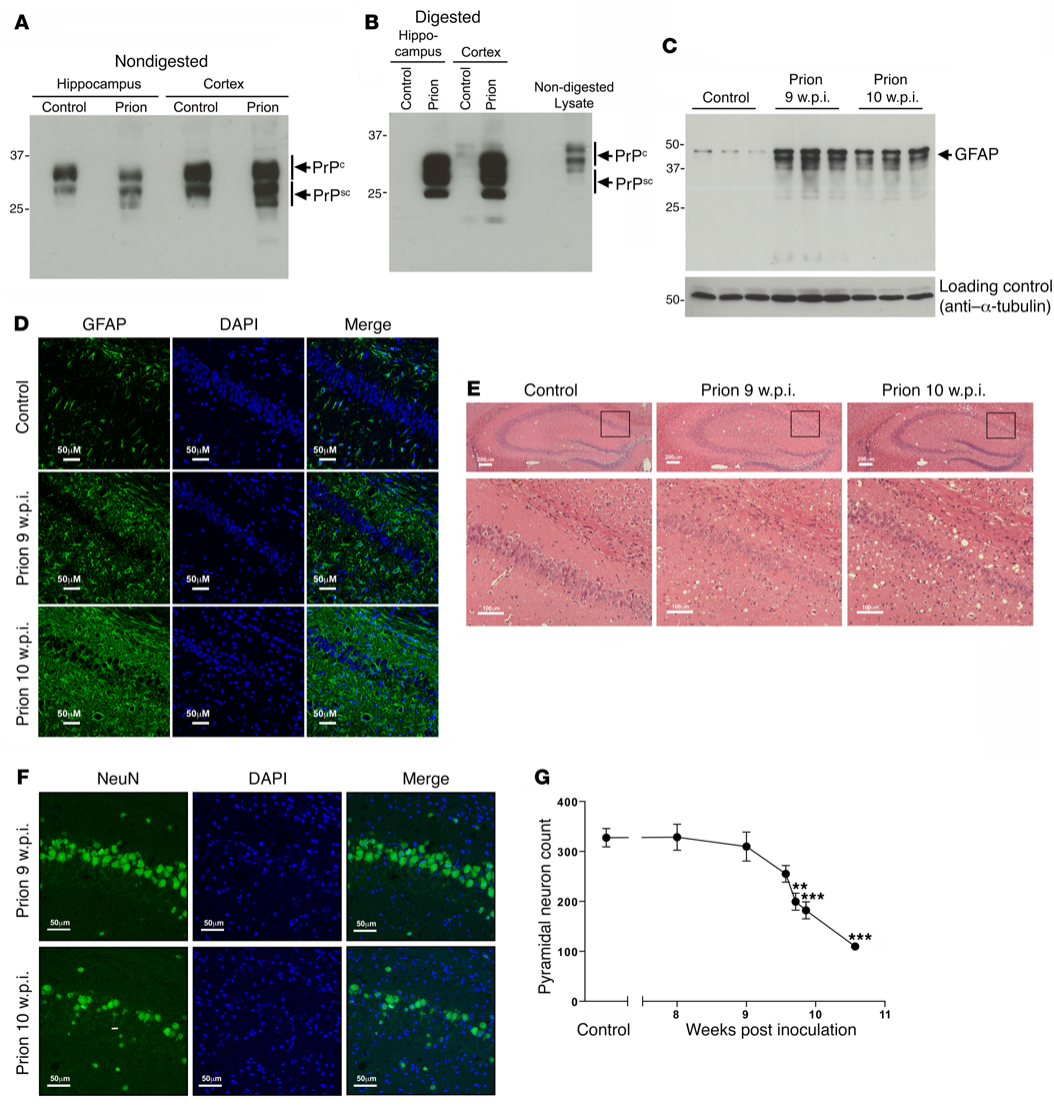
Mouse prion disease is associated with the accumulation of misfolded prion protein, astrogliosis, spongiosis, and hippocampal neuronal loss. (**A**) Lysates from control or prion-infected (10 w.p.i.) mouse hippocampus and cortex were probed in Western blots with an antibody that detected both cellular (PrP^c^) and misfolded (PrP^Sc^) prion protein. The presence of misfolded PrP^sc^ is evident by lower molecular weight species in the prion-infected lysates. (**B**) Lysates from **A** were treated with proteinase K before probing in Western blots for prion protein. (**C**) Astrogliosis during prion disease was determined in Western blots of lysates prepared from control mice or mice 9 and 10 w.p.i. and probed with glial fibrillary acidic protein (GFAP), a marker for astrocytes. (**D**) Immunohistochemical staining of the hippocampal CA1 region probed with anti–glial fibrillary acidic protein antibody (green) to determine the level of astrogliosis. The nuclei were stained blue with DAPI. Scale bars: 50 μm. (**E**) Spongiosis in prion-infected hippocampi (upper panels) and CA1 region (lower panels) was visualized by H&E stain of mouse hippocampus from paraformaldehyde-fixed mouse brain from control mice injected with NBH (control), prion-infected mice 9 w.p.i., and prion-infected mice 10 w.p.i. Scale bars: 200 μm (upper panels); 100 μm (lower panels). (**F**) Determination of neuronal loss of pyramidal neurons in the CA1 region of the hippocampus of mice 9 and 10 w.p.i. was determined by immunohistochemical staining of neuronal cell bodies using antibodies to NeuN (green). The nuclei were stained blue with DAPI. Scale bars: 50 μm. (**G**) Quantification of NeuN staining in the CA1 of mice at various stages of prion disease. Data are shown as mean ± SEM. *n* = 3 mice, 3 sections per mouse. **P < 0.01; ***P < 0.001, 1-way ANOVA. Blots and images shown are representative of at least 3 independent experiments.

**Figure 3:**
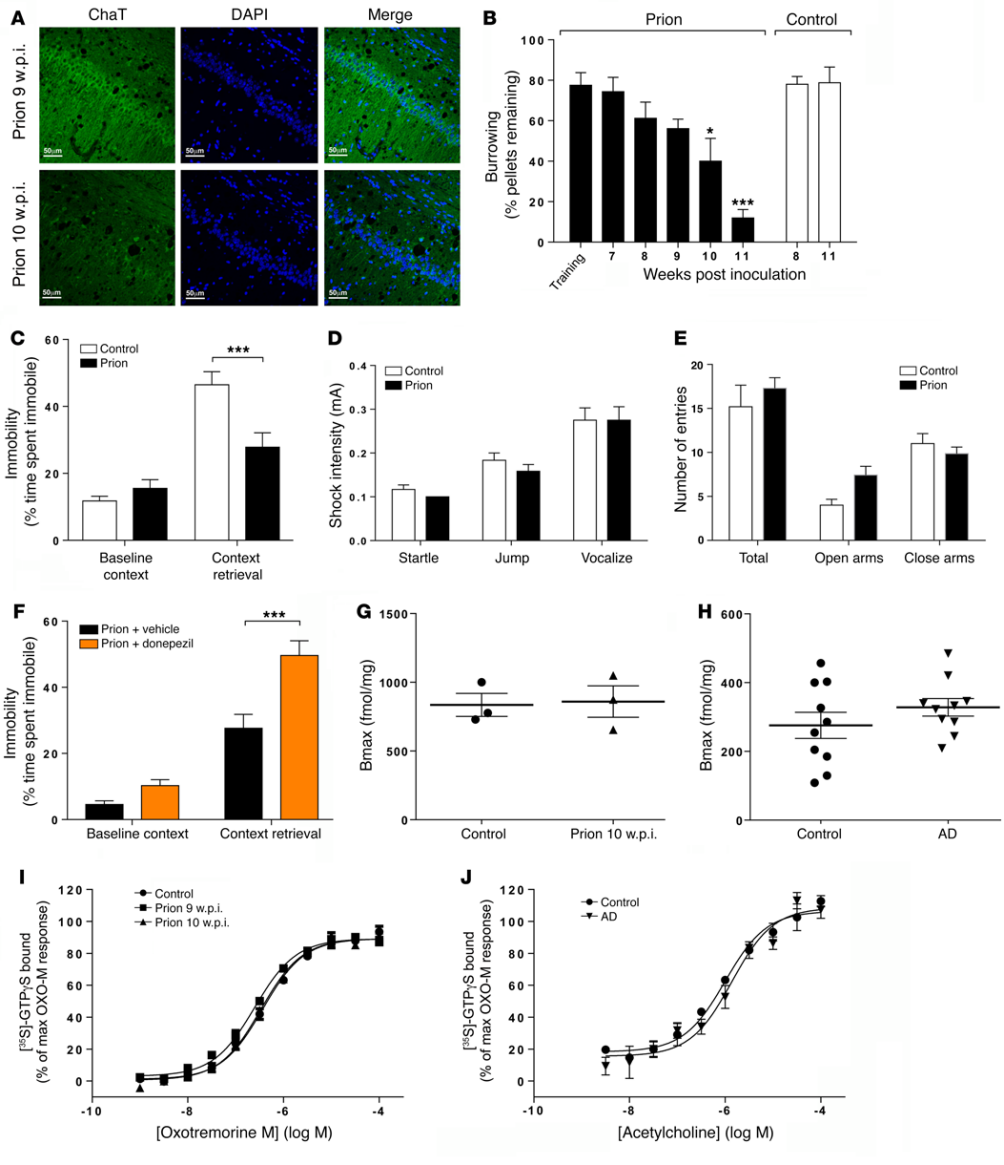
Prion disease is associated with a disruption in hippocampal cholinergic innervation, a deficit in learning, and memory rescued by donepezil, while maintaining muscarinic receptor expression and signaling. (**A**) Cholinergic innervation of the hippocampus was assessed by ChAT (green) immunostaining of the CA1 region of the hippocampus in mice at 9 and 10 w.p.i. Data shown are representative of 3 individual mice per group. Scale bars: 50 μm. (**B**) Burrowing response of control and prion-infected mice. *n* = 4–9 mice. *P < 0.05; ***P < 0.001, 1-way ANOVA. (**C**) Fear-conditioning response of control and prion-infected mice at 9–10 w.p.i. *n* = 19 mice per group. ***P < 0.001, 2-way ANOVA with Sidak’s multiple comparison test. (**D**) Pain threshold response of control and prion-infected mice at 9–10 w.p.i. *n* = 6 mice per group. Unpaired Student’s *t* test. (**E**) Anxiety levels of control and prion-infected mice at 9–10 w.p.i. were assessed by elevated plus maze. *n* = 6 mice per group. Unpaired Student’s *t* test. (**F**) Fear-conditioning response of prion-infected mice (9–10 w.p.i.) treated with vehicle or donepezil (0.5 mg/kg) 60 minutes before training. *n* = 9 (vehicle); *n* = 15 (donepezil). ***P < 0.001, 2-way ANOVA with Sidak’s multiple comparison test. (**G**) Determination of the total muscarinic receptor population by [^3^H]-NMS binding to hippocampal membranes prepared from control or prion-infected mice (10 w.p.i.). Nonspecific binding was determined by the addition of atropine (1 μM). Data are expressed as fmol/mg protein (*n* = 3). Bmax, maximal binding capacity. (**H**) Total [^3^H]-NMS binding to membranes prepared from the frontal cortex of control or AD patients. *n* = 10. (**I**) Stimulation of [^35^S]-GTPγS binding to membranes prepared from control or prion-infected mice (9–10 w.p.i.) in response to oxotremorine-M. Data shown are increases in [^35^S]-GTPγS binding over basal; mean pEC_50_ values of 6.45 ± 0.03, 6.63 ± 0.01, and 6.50 ± 0.04, respectively (*n* = 3). (**J**) [^35^S]-GTPγS binding to membranes prepared from the frontal cortex of control or AD patients in response to acetylcholine. Data are the percentage of the maximal [^35^S]-GTPγS binding stimulated by oxotremorine-M. Mean pEC_50_ values of 6.00 ± 0.09 and 5.86 ± 0.11, respectively (*n* = 3).

To establish the impact of M1 mAChR ligands on learning and memory in neurodegenerative disease, we used prion-diseased mice that had progressive neurodegenerative disease with mechanistic, behavioral, and neuroanatomical correlates to human and animal disorders (36, 37). Consistent with previous studies (38, 39), inoculation of mice (Tg37 hemizygous) with Rocky Mountain Laboratory (RML) prion–infected brain homogenate resulted in the accumulation of misfolded insoluble prion protein (PrP^Sc^) in the hippocampus and cortex (Figure 2A) that was insensitive to proteinase K digestion (Figure 2B). There was also a notably higher expression of prion protein in the cortex compared with the hippocampus (Figure 2B). Prion-infected brains also showed significant astrogliosis (Figure 2, C and D) and spongiosis (Figure 2E) as well as progressive neuronal loss in the CA1 region of the hippocampus from 9 weeks post inoculation (w.p.i.), as evidenced by histological staining of the hippocampus (Figure 2E) and a reduction in the staining of the marker for neuronal cell bodies, NeuN (Figure 2, F and G). Importantly, the loss of neuronal cell bodies was also associated with a disruption of cholinergic innervation in the hippocampus, as evidenced by a fragmentation of neurons staining positively for choline acetyltransferase (ChAT), a marker of cholinergic neurons (Figure 3A). This correlated with a deficit in burrowing behavior (Figure 3B), as previously reported (24, 26), but also a deficit in contextual fear–conditioning learning and memory (Figure 3C), whereas levels of locomotion, anxiety, and pain thresholds were not affected (Figure 3, D and E). It is important to note that these behavioral tests were conducted at a time point (9 to 10 w.p.i.) at which the mice showed no physical signs of disease. Physical symptoms of mouse prion disease, including ataxia, impairment of righting reflex, dragging of limbs, and sustained hunched posture, occur later in disease progression (from 12 w.p.i.).

The current frontline treatment of AD is administration of AChE inhibitors, such as the clinically approved drug donepezil, which increases cholinergic transmission by preventing acetylcholine degradation. Administration of donepezil (0.5 mg/kg) 60 minutes prior to fear-conditioning training significantly augmented learning and memory in prion-infected mice (Figure 3F), indicating that the memory loss in prion-diseased mice was mechanistically linked to a deficit in cholinergic transmission — a feature common to memory loss observed in AD.

A further correlation between murine prion disease and AD was revealed through the investigation of the expression and signaling status of hippocampal muscarinic receptors. The expression levels of mAChRs were determined by radioligand binding and found to be unchanged in prion disease up to 10 w.p.i. (Figure 3G and Supplemental Figure 3, A and B), as was the expression of M1 mAChRs (Supplemental Figure 3C), which have previously been reported to have a postsynaptic localization (40). Similarly, we found that in the AD patient samples, the mAChR population was not significantly changed when compared with nondiseased controls (Figure 3H). The coupling of mAChRs to cognate G proteins, as determined by receptor-mediated [^35^S]-GTPγS binding, was found to be the same in hippocampal membranes prepared from control and prion-infected mice (Figure 3I). Importantly, the coupling of mAChRs in cortical membranes from AD patients was also the same as in nondiseased controls (Figure 3J). Thus, our data indicate that cholinergic transmission is defective in prion disease due to a disruption of the presynaptic cholinergic input, while the postsynaptic cholinergic signaling apparatus remains intact — features that murine prion disease shares with AD, as evidenced in our study and that of others (41, 42).

### Targeting mAChRs restores the memory deficit in prion disease.

**Figure 4:**
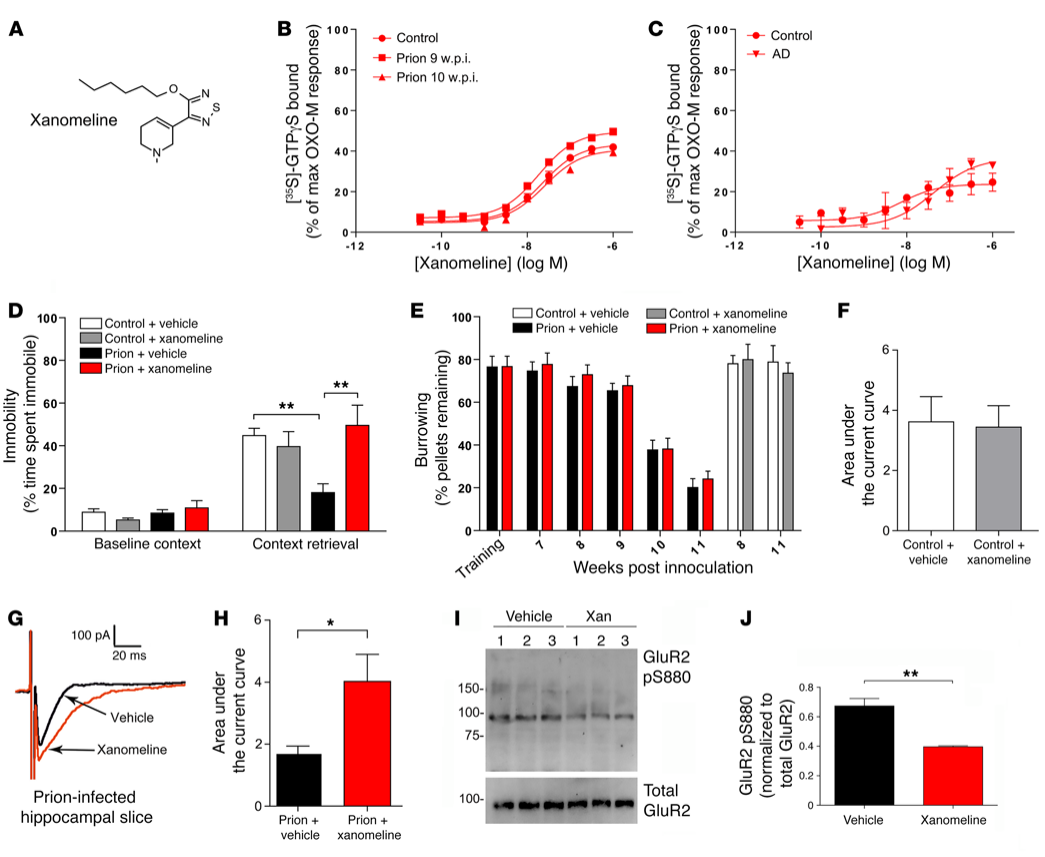
The orthosteric mAChR agonist xanomeline restores the learning and memory deficit in prion-infected mice. (**A**) Chemical structure of xanomeline. (**B**) [^35^S]-GTPγS binding to membranes prepared from control or prion-infected mice (9–10 w.p.i.) in response to xanomeline are expressed as a percentage of the maximal response observed with oxotremorine-M. Mean pEC_50_ for xanomeline on control membranes = 7.67 ± 0.04, prion 9 w.p.i. membranes = 7.73 ± 0.06, and prion 10 w.p.i. membranes = 7.65 ± 0.13. *n* = 3. (**C**) [^35^S]-GTPγS binding to membranes prepared from the frontal cortex of control or AD patients in response to xanomeline. Data are expressed as the percentage of the maximal [^35^S]-GTPγS binding stimulated by oxotremorine-M. Mean pEC_50_ values of 8.14 ± 0.28 (control) and 7.34 ± 0.36 (AD). *n* = 3. (**D**) Fear-conditioning response of control and prion-infected mice following administration of vehicle or xanomeline (5 mg/kg) 30 minutes prior to training and retrieval. *n* ≥ 6. Statistical analysis by 1 -way ANOVA. **P < 0.01. (**E**) Burrowing response of control and prion-infected mice following administration of vehicle or xanomeline (5 mg/kg) 30 minutes before each burrowing session (from 7 w.p.i.). (**F**, **G**, and **H** ) AUC of AMPA receptor–mediated currents before and after treatment with xanomeline in control (**F**, *n* = 10) and prion-infected (**G** and **H**, *n* = 12) hippocampi. *P < 0.05, paired Student’s *t* test. Also shown in **G** are representative traces of paired whole cell CA1 glutamatergic current recordings in vehicle-treated (black) and xanomeline-treated (100 nM) (red) hippocampal slices of a prion-infected mouse. (**I**) Western blot of hippocampal lysates prepared from prion-infected mice treated with vehicle or xanomeline (Xan, 5 mg/kg) and probed with an antibody that detects phospho-S880 of GluR2 AMPA receptor subunits (total GluR2 was used as a loading control). (**J**) Quantification of **I**. *n* = 3. **P < 0.01, paired Student’s *t* test. Data are shown as mean ± SEM.

To test the hypothesis that targeting mAChRs in prion disease can have an impact on the learning and memory deficit, we used the M1/M_4_-preferring mAChR orthosteric ligand xanomeline (43, 44) (Figure 4A), which acts as a partial agonist for G protein coupling to mAChRs in the hippocampus of control and prion-infected mice (Figure 4B). As further evidence of the similarities in pharmacological agent responsiveness in prion disease and AD, we found that xanomeline also acts as a partial agonist in human cortical samples in a manner that is not affected in AD patient samples (Figure 4C). Our brain and plasma exposure data are consistent with previous pharmacokinetic (45) data and show that xanomeline has good bioavailability in murine hippocampus 30 minutes after i.p. administration (Supplemental Table 1). In behavioral studies, administration of xanomeline (5 mg/kg) 30 minutes before contextual fear–conditioning training and 30 minutes before context retrieval resulted in a complete restoration of the contextual fear–conditioning deficit in prion-infected mice (Figure 4D). This effect appeared specific to hippocampal responses associated with learning and memory, since the burrowing response of prion-infected mice, a hippocampal-based innate behavior not associated with memory (46, 47), was not affected (Figure 4E).

It is well established that hippocampal glutamatergic transmission is closely associated with learning and memory (48). We therefore investigated the relationship between mAChR activation and glutamate transmission in prion disease. Electrophysiological assessment of hippocampal slices determined that the glutamatergic α-amino-3-hydroxy-5-methylisoxazolepropionate (AMPA) receptor current in hippocampal CA1 pyramidal neurons was not affected by xanomeline treatment in control samples, as indicated by analysis of the AUC (Figure 4F). In contrast, xanomeline significantly upregulated the AMPA receptor current in the hippocampi of prion-infected mice (Figure 4, G and H), an effect that can be attributed entirely to an increase in the decay time, since no change in evoked current amplitude was detected (Supplemental Figure 4, A and B). The upregulation of AMPA receptor–mediated glutamatergic transmission in response to xanomeline in prion disease correlated with a decrease in the phosphorylation status of the AMPA subunit GluR2 at S880 (Figure 4, I and J). This dephosphorylation event has previously been shown to promote the interaction between GluR2 and PICK1 and thereby regulate GluR2 internalization, intracellular trafficking, and long-term depression (LTD) (49–53). Interestingly, the action of xanomeline was selective for GluR2, since phosphorylation of GluR1 at S831 and S845 was not affected (Supplemental Figure 5).

### Highly selective M1 mAChR PAMs rescue the memory deficit in prion disease.

**Figure 5:**
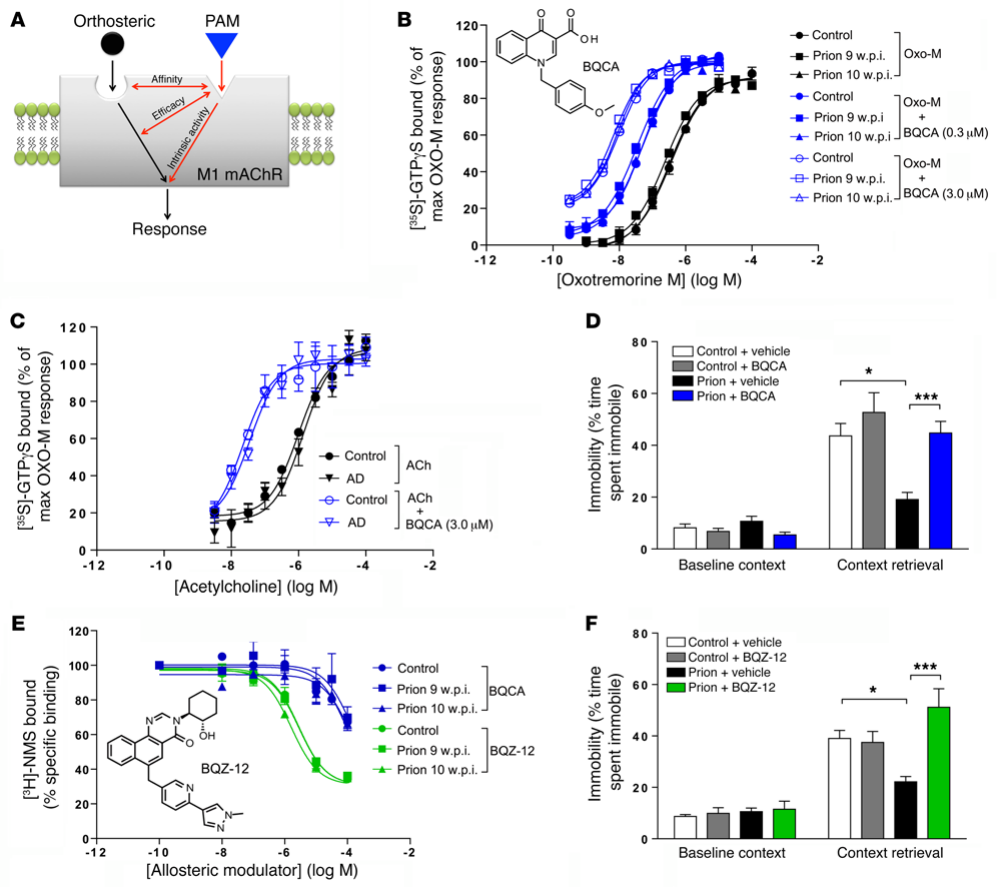
PAMs of the M1 mAChR rescue the fear-conditioning learning and memory deficit in prion-infected mice. (**A**) Schematic summarizing the 3 possible effects of an allosteric modulator, namely, modulation of orthosteric ligand affinity, signaling efficacy, and/or direct activation. (**B**) BQCA (inset; chemical structure of BQCA) causes equivalent leftward shifts (black arrow) of the oxotremorine-M (Oxo-M) [^35^S]-GTPγS-assay concentration-response curve and displays intrinsic activity (red arrow) in hippocampal membranes derived from control and prion-infected mice (9 and 10 w.p.i.). Data are shown as mean ± SEM. *n* = 3. (**C**) Acetylcholine-stimulated [^35^S]-GTPγS binding to membranes prepared from the frontal cortex of control or AD patients in the absence and presence of BQCA (3 μM). Data are expressed as the percentage of the maximal [^35^S]-GTPγS binding stimulated by oxotremorine-M. Mean ± SEM. *n* = 3. (**D**) Fear-conditioning response of control and prion-infected mice following administration of vehicle or BQCA (15 mg/kg) 30 minutes prior to training. Mean ± SEM. *n* = 6–18. *P < 0.05; ***P < 0.001, 1-way ANOVA. (**E**) Radioligand competition binding between [^3^H]-NMS (~0.3 nM) and increasing concentrations of BQCA or BQZ-12 (inset, chemical structure of BQZ-12) in hippocampal membranes from control and prion-infected mice (9 and 10 w.p.i.). *n* = 3–4. The affinities (pKi) of BQCA and BQZ-12 at hippocampal membranes from prion-diseased mice (10 w.p.i.) were 6.15 ± 0.08 and 4.25 ± 0.12, respectively. Data are shown as mean ± SEM. *n* = 3. (**F**) Fear-conditioning response of control and prion-infected mice following administration of vehicle or BQZ-12 (1.5 mg/kg) 30 minutes prior to training. Data are shown as mean ± SEM. *n* = 12–19. *P < 0.05; ***P < 0.001, 1-way ANOVA.

In contrast to orthosteric ligands, such as xanomeline, M1 mAChR PAMs provide exquisite selectivity by binding to nonconserved regions of the M1 mAChR (16, 17) and act by modulating the action of the natural ligand acetylcholine (Figure 5A), thereby maintaining the spatiotemporal features of cholinergic transmission (54). BQCA (structure in Figure 5B) has previously been described as a highly selective M1 mAChR PAM that markedly increases the affinity of orthosteric agonists at the M1 mAChR (30–32). Here, we demonstrate that BQCA acts as a PAM in hippocampal membranes derived from control, 9 and 10 w.p.i. prion-infected mice, where it augments the action of an orthosteric agonist (in this case oxotremorine-M) as evidenced by the progressive leftward shift in the oxotremorine-M [^35^S]-GTPγS concentration–response curve with increasing concentrations of BQCA (Figure 5B). Importantly, the PAM activity of BQCA is also evident in membranes derived from both AD patient samples and nondisease controls, indicating that BQCA acts to enhance the activity of orthosteric agonists in the context of both murine prion disease and AD (Figure 5C). The maximal effect of BQCA in prion disease and AD was to enhance the potency of agonists by approximately 100-fold (see negative logarithm of the half-maximal effective concentration [pEC_50_] values in Supplemental Table 2). In addition, BQCA displayed some direct intrinsic agonist activity in mouse hippocampal membranes, a response evident at high BQCA concentrations (i.e., 3 μM) (Figure 5B), identifying this compound as a PAM agonist in this tissue. This intrinsic activity can entirely be attributed to the action of BQCA at hippocampal M1 mAChRs, since there is a complete absence of the BQCA [^35^S]-GTPγS response in membranes derived from M1-KO mice (Supplemental Figure 6). Interestingly, no intrinsic activity of BQCA was noted in the AD patient samples, highlighting that in the human tissues, the compound behaved as a pure PAM.

Previous pharmacokinetic studies have demonstrated that BQCA reaches maximal brain exposure after 30 to 60 minutes and remains stable for approximately 4 hours after i.p. administration (19). Our studies are consistent with these data and show that BQCA (15 mg/kg; i.p.) administered 30 minutes prior to contextual fear–conditioning training resulted in brain exposure at concentrations at which BQCA would be expected to show only PAM activity (Supplemental Table 1). Thus, any in vivo effects at this exposure of BQCA in the mice would not be attributable to any potential intrinsic activity of BQCA (i.e., “PAM-agonist” activity), but only attributable to its PAM activity. Under these conditions, single administration of BQCA prior to training resulted in a complete restoration of the contextual fear–conditioning learning and memory deficit observed in prion-infected mice at 9 to 10 w.p.i. (Figure 5D). Electrophysiological evaluation of the action of BQCA revealed that, similarly to xanomeline, BQCA was able to restore AMPA-dependent glutamate transmission in prion-infected hippocampal slices (Supplemental Figure 7).

A recent chemistry program developed an M1 mAChR PAM, BQZ-12 (structure in Figure 5E), which is structurally related to BQCA, but shows higher functional potency based largely on a substantial improvement in allosteric site–binding affinity (33). We established that the affinity for BQZ-12 at the M1 mAChR in prion-infected hippocampus was approximately 40-fold greater than that of BQCA (Figure 5E). Administration of BQZ-12, at a dose 10-fold lower than that previously used for BQCA, 30 minutes prior to contextual fear–conditioning training of prion-infected mice (9 to 10 w.p.i.) resulted in complete restoration of the learning and memory deficit in these mice (Figure 5F).

**Figure 6:**
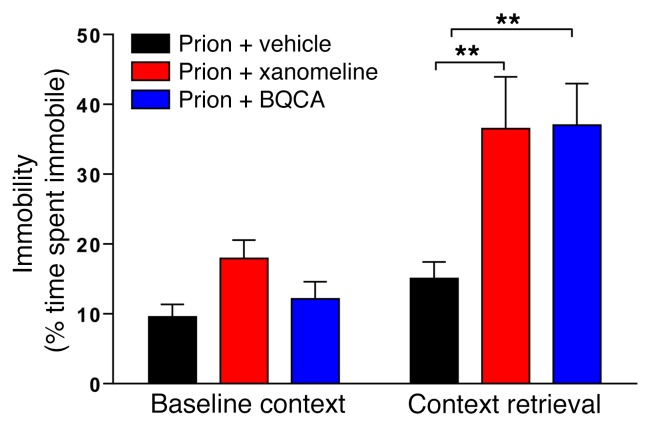
Orthosteric agonists and PAMs of the M1 mAChR are equivalently efficacious in restoring fear-conditioning learning and memory deficit in prion-infected mice. Fear-conditioning response of prion-infected mice following administration of vehicle, xanomeline (5 mg/kg), or BQCA (15 mg/kg) 30 minutes prior to training (*n* = 13–18). Data are shown as mean ± SEM. **P < 0.01, 1-way ANOVA.

Hence, our results indicate that selective enhancement of hippocampal M1 mAChR activity via PAMs is able to restore the learning and memory deficit in prion disease to an extent equal to that observed with the clinically validated muscarinic orthosteric ligand xanomeline. To test this notion directly, we conducted a head-to-head comparison of the efficacy of xanomeline and BQCA and found that indeed they both showed similar efficacy in the augmentation of the memory response in prion-diseased mice (Figure 6).

### Daily treatment of prion-diseased mice with BQCA prolonged survival.

**Figure 7:**
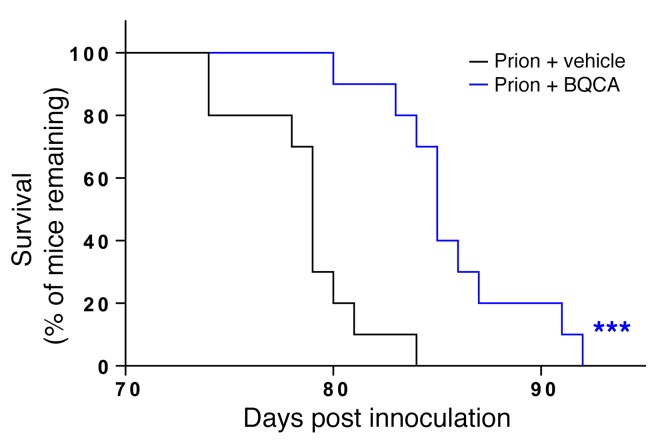
PAMs of the M1 mAChR significantly increase survival in prion-diseased mice. Kaplan-Meier survival plots for prion-infected mice treated with vehicle (5% glucose; *n* = 10; black line) or BQCA (15 mg/kg; *n* = 10; blue line) daily from 7 w.p.i. ***P < 0.001, Gehan-Breslow-Wilcoxon test.

The selective nature of M1 mAChR PAMs and the mechanism of action that maintains the spatiotemporal aspects of endogenous acetylcholine transmission has led us to posit that M1 mAChR PAMs will have lower adverse effects than orthosteric muscarinic receptor agonists (e.g., xanomeline) and AChE inhibitors (donepezil). This notion is supported by our study of the adverse effects of escalating doses of xanomeline, donepezil, BQCA, and BQZ-12, where we saw no evidence of adverse responses to BQCA or BQZ-12 at doses in excess (2×) of those necessary for efficacy in learning and memory responses (Supplemental Table 3). In contrast, there were significant adverse reactions to both xanomeline and donepezil at doses twice that required to restore learning and memory. The lack of adverse reactions to BQCA prompted us to test the possibility that continued daily dosing of prion-infected mice with BQCA might modify disease progression. Daily dosing of prion-infected mice (15 mg/kg) from 7 w.p.i (a time point just prior to the appearance of unfolded prion protein; ref. 26) resulted in no adverse drug reactions during the time course of the experiment (up to 13 w.p.i.). Also, there was no significant change in the body mass of mice injected daily with either vehicle or BQCA from 7 w.p.i. up to the onset of clinical disease (Supplemental Figure 8). Importantly, the mice dosed with BQCA showed a significant delay in the onset of confirmatory scrapie diagnosis (Figure 7), data that support the notion that targeting the M1 mAChR with selective PAMs affects not only the symptoms, but also the progression of neurodegenerative disease.

## Discussion

M1 mAChRs are considered viable targets in human neurodegenerative disease (7, 14). However, drug development has been unsuccessful due to the problems of generating sufficiently selective orthosteric M1 mAChR agonists. In the current study, we tested the notion that the barriers presented by subtype selectivity could be overcome by the use of highly selective M1 mAChR PAMs. The question, however, is whether PAMs that operate by enhancing the action of the endogenous agonist acetylcholine would show sufficient activity or positive cooperativity to have an impact on memory and learning in neurodegenerative disease associated with cholinergic dysfunction. In order to test this hypothesis, we used mice with prion disease, which we show here to be a neurodegenerative disease, with mechanistic, behavioral, and neuroanatomical correlates to animal and human disorders (36, 37). In particular, there are disruptions of hippocampal cholinergic innervation and associated memory loss similar to those described in AD patients (41). Furthermore, we show that postsynaptic muscarinic receptor expression and signaling, including that of M1 mAChR, remains intact in prion disease as it does in AD patients (42). The correlation between prion disease and AD was further extended by the finding that the clinically approved AChE inhibitor donepezil restored the learning and memory deficit in prion disease, indicating that diminished cholinergic transmission was mechanistically linked to the memory loss observed in prion disease.

We determined that M1 mAChR PAMs, BQCA (30–32), and BQZ-12 (33), which show high levels of selectivity and functional cooperativity at the M1 mAChR, also retain these pharmacological properties at hippocampal M1 mAChRs in the context of both prion disease and AD patient samples. Importantly, pharmacokinetic analysis revealed that the brain exposure levels of BQCA in mice were such that this ligand would act as a pure PAM at these concentrations. Moreover, only PAM activity was noted in the human AD patient samples. Collectively, therefore, any in vivo activity of BQCA is likely to reflect pure PAM activity and not a mixture of PAM and intrinsic agonist (i.e., PAM-agonist) activity. Administration of both BQCA and BQZ-12 completely restored the learning and memory response in prion-diseased mice. In so doing, these PAMs mimicked the response of the orthosteric M1/M_4_-preferring agonist xanomeline, which was previously shown to restore some of the behavioral disturbances associated with AD and schizophrenia (12, 13). Hence, the level of functional cooperativity displayed by M1 mAChR PAMs used in this study was sufficient to restore the learning and memory response of prion-diseased mice, where defective memory was associated with cholinergic dysfunction. The clinical implication of these findings is that M1 mAChR PAMs might similarly show sufficient functional cooperativity to enhance defective cholinergic transmission, and thereby restore functions such as learning and memory, in human neurodegenerative diseases that show a deficit in cholinergic transmission, such as AD.

Since synaptic plasticity mediated by AMPA receptor activity has long been associated with learning and memory (55), we investigated whether there was any correlation between mAChR-mediated rescue of learning and memory in prion disease and hippocampal glutamatergic transmission. We determined that, in prion disease, AMPA receptor–mediated glutamatergic transmission was upregulated by enhancing M1 mAChR activity via direct activation of the receptor with xanomeline or by functional cooperativity between an M1 mAChR PAM (BQCA) and the endogenous ligand acetylcholine. The effect of M1 mAChR activation on glutamate transmission was associated with a change in the phosphorylation of GluR2 at S880 — a phosphorylation event shown previously to promote GluR2 internalization and mediate LTD (49–53). Our observations, therefore, suggest a mechanism whereby M1 mAChR activation, through orthosteric or allosteric ligands, can have an impact on learning and memory deficits in prion disease by promoting dephosphorylation of GluR2 at S880, thereby promoting maintenance of AMPA receptors at synaptic membranes with a subsequent strengthening of AMPA receptor–mediated transmission. It is likely that this mechanism would run in parallel with other effects of M1 mAChR activation observed here in prion-diseased hippocampus, such as enhanced hippocampal M1 mAChR signaling.

M1 mAChR PAMs have been suggested to hold an advantage over orthosteric muscarinic ligands as drug candidates due to their higher degree of M1 AChR selectivity. This selectivity is mediated by a mechanism that maintains the spatiotemporal signaling of the endogenous ligand acetylcholine and/or by acting in a saturable manner, resembling the characteristic “ceiling” effect of acetylcholine (16, 17). Collectively, these features suggest that M1 mAChR PAMs might show clinical efficacy with reduced adverse responses. Certainly, the relatively nonselective nature of AChE inhibitors has resulted in dose-limiting adverse effects. That PAMs might have a superior safety profile compared with that of AChE inhibitors and orthosteric ligands is supported by our study, where no significant adverse responses to M1 mAChR PAMs were observed at concentrations that exceeded those necessary for restoration of learning and memory. The high level of tolerability to M1 mAChR PAMs in our study allowed us to test whether prolonged daily dosing with BQCA could have any impact on prion disease progression. We show here that continued dosing significantly reduced the onset of clinical signs of prion disease, thereby extending the life span of prion-diseased mice.

Mechanistically, BQCA (and BQZ-12) act by increasing the affinity of M1 mAChRs for acetylcholine by approximately 100-fold (33, 35, 56). There is no evidence that these M1 mAChR PAMs can also increase the intrinsic efficacy of acetylcholine at M1 mAChRs over and above any effects on acetylcholine-binding affinity. In addition, the in vivo concentrations of unbound brain BQCA are at levels where BQCA would not be expected to show any intrinsic agonist activity in its own right, but would rather act as a pure PAM. Thus, the in vivo responses (on both memory and disease progression) likely reflect actions on the binding of acetylcholine to M1 mAChRs rather than any direct stimulatory actions on the receptor itself or changes in its inherent signaling properties. Whereas this mechanism of action likely underlies the potential safety profile of the PAMs used here (see above), one clinical implication is that, as disease progresses, the cholinergic deficit may fall to a level where even an enhancement of acetylcholine affinity by 100-fold will not be sufficient to restore functional responses such as learning and memory. In these circumstances, it might also be necessary to consider PAMs that also possess either greater intrinsic activity and/or cooperativity at the level of agonist-receptor signal transduction properties to restore function in cases of advanced neurodegenerative disease associated with cholinergic deficit.

In conclusion, our study provides evidence that activating M1 mAChRs can not only restore memory loss in neurodegenerative disease in a manner that is associated with an upregulation of glutamatergic transmission, but can also be disease modifying, slowing the onset of severe clinical symptoms. This opens the door to the prospect that M1 mAChR PAMs might have not only a clinical impact on the symptomatic treatment of memory loss in neurodegenerative diseases showing defective cholinergic transmission, such as AD, but may also have the potential to slow disease progression.

## Methods

### Mouse maintenance and diet.

Mice were fed ad libitum with a standard mouse chow. The mAChR knockout mice were backcrossed for at least 10 generations onto the black C57BL6/NTAC background. The Tg37 mouse line that overexpresses mouse prion protein has been described previously (26).

### Drug administration and pharmacokinetics.

Compounds were administered via i.p. injection 30 minutes prior to tissue/blood collection. Following this, mice were anesthetized with 3% isoflurane (2 l/min O_2_), and blood was collected by cardiac puncture of the left ventricle. Blood was immediately transferred to EDTA tubes and centrifuged at 1,000 *g* for 10 minutes at 4°C; supernatant was collected and frozen. Brains from each mouse were also dissected and snap-frozen on dry ice.

Brain samples were homogenized in 3 volumes of methanol/water (1:4, v/v) by weight. A 25 μl aliquot of each study sample, calibration standard, and control sample were added to a 96-well plate, then mixed with 180 μl of acetonitrile/methanol (1:1, v/v) containing internal standard. After mixing, the samples were centrifuged, and the resulting supernatants were diluted 12.5-fold with methanol/water (1:1, v/v) prior to analyzing 10 μl aliquots by liquid chromatography–MS/MS (LC-MS/MS).

The sample extracts were analyzed with an Applied Biosystems/MDS Sciex API 4000 triple quadrupole mass spectrometer. The analytes were chromatographically separated using a ThermoHypersil Betasil C18 2 × 20 mm 5-micron Javelin HPLC column, with a gradient LC system composed of water/trifluoroacetic acid/1 M ammonium bicarbonate, (1000:4:1, v/v) (mobile phase A), and acetonitrile/trifluoroacetic acid/1 M ammonium bicarbonate, (1000:4:1, v/v) (mobile phase B). Data were acquired and processed with Applied Biosystems/MDS Sciex Analyst software (version 1.4.2).

The unbound fraction of drug in brain was estimated using fast gradient elution LC-MS/MS to estimate the percentage of compound bound to brain over a 4.5-hour incubation period at 37°C while undergoing orbital shaking. The assay was performed using a HT dialysis micro-equilibrium device, using dialysis membrane strips (molecular weight cut off, 12–14 k). At time 0, a sample of brain homogenate was taken, and samples were taken from both the protein side and buffer side of the membrane after 4.5 hours of incubation. Drug concentrations were measured as described previously (57). Fraction unbound was calculated by dividing the concentration of the buffer side by the concentration of the protein side.

### Prion infection of mice.

Tg37 hemizygous mice were inoculated by intracerebral injection into the right parietal lobe with 1% brain homogenate of RML prions aged 3 to 4 weeks as described previously (26). Control mice received 1% normal brain homogenate (NBH).

### Survival studies.

Tg37 mice were inoculated with NBH or RML prions as above. Mice were treated (i.p.) with vehicle (5% glucose) or BQCA (15 mg/kg) daily from 7 w.p.i. Animals were culled when they developed clinical signs of scrapie; prion-infected mice were scored according to the appearance of recognized early indicator and confirmatory signs of prion disease. Early indicator signs included piloerection, sustained erect ears, erect penis, clasping of hind legs when lifted by tail, rigid tail, unsustained hunched posture, mild loss of coordination, and being subdued. Confirmatory signs of prion disease included ataxia, impairment of righting reflex, dragging of limbs, sustained hunched posture, and significant abnormal breathing. The presence of 2 early indicator signs plus 1 confirmatory sign or of 2 confirmatory signs alone was used to diagnose clinical disease.

### Fear-conditioning learning and memory test.

For behavioral testing of C57BL6/NTAC or M1-KO mice, 8- to 12-week-old male mice were used. For prion-infected mice and the relevant control mice, behavioral experiments were conducted on male mice between 9 and 10 w.p.i. with NBH or RML prions prior to the appearance of clinical symptoms (listed above). Mice were acclimatized to the behavioral room for at least 2 hours prior to the test. For fear conditioning, mice were placed in the conditioning chamber (Stoelting ANY-maze Fear Conditioning System) and, after a 2-minute adaptation period, received 3 tone/foot shock pairings where the foot shock (unconditioned stimulus [US]; 2 seconds; 0.4 mA) always coterminated with a tone (conditioned stimulus [CS]; 2.8 kH; 85 dB; 30 seconds). The CS-US pairings were separated by 1-minute intervals. After completion of training, the mice remained in the conditioning chamber for 1 minute and were then returned to their home cages. The next day, mice were placed back in the conditioning chamber, and time spent immobile was recorded for 3 minutes to assess context-dependent learning. Data were analyzed using ANY-maze software.

### Pain threshold.

The mice were placed on the grid floor of the conditioning chamber (described above for fear conditioning) and were given 2-second foot shocks of increasing intensity (0.10 to 0.4 mA) at 10-second intervals. The level of the electric current needed to elicit startle, running/jumping, and vocalization responses was determined. All animals were foot shock naive before the experiment and were not used for any subsequent tests.

### Burrowing.

Assessment of burrowing was conducted on mice from 7 w.p.i. The burrowing test involved mice being placed into individual cages with a plastic cylinder filled with 140 g of food pellets. Food remaining in the cylinders after 2 hours was weighed and the amount displaced (“burrowed”) was calculated. Prior to the burrowing test, mice were placed in the burrowing cage for a 2-hour period. On the following day, mice received vehicle or xanomeline (5 mg/kg) via i.p. injection 30 minutes prior to the burrowing test. This was then repeated on a weekly basis.

### Open field.

This test was used to analyze general locomotor activity levels. The mice were placed into a clear Perspex square arena (50 × 50 cm), and activity was tracked for a 10-minute period using ANY-maze software.

### Elevated plus maze.

The elevated plus maze apparatus consisted of 4 nontransparent arms (50 × 10 cm): 2 enclosed arms (with black walls of 30-cm height) that formed a cross shape with 2 open arms opposite each other. The open arms were dimly illuminated. Mice were placed at the center of the maze facing an open arm. Mice were tracked for 5 minutes, and their tendency toward dark, enclosed spaces versus the open spaces was used as a measure of anxiety. The number of entries of the animal from the central platform into the enclosed or open arms was counted, and data were recorded using the ANY-maze software.

See Supplemental Information for methods for the following: generation of M1 mAChR phospho-S228–specific antiserum, radioligand binding assays, [^35^S]-GTPγS assay, electrophysiology, immunocytochemistry, and immunoblotting.

### Statistics.

Statistical analyses were performed using 2-tailed Student’s *t* test, 1-way ANOVA, or 2-way ANOVA. Significance was defined as *P* < 0.05. All statistical tests were performed using GraphPad Prism software.

### Study approval.

All animal work conformed to the United Kingdom Home Office regulations. All procedures (both nonregulated and regulated) were conducted under a Home Office project licence awarded to Andrew Tobin under the Animals (Scientific Procedures) Act 1986. Human brain tissue from post mortem healthy and AD patients was provided to Eli Lilly from the Oregon Alzheimer’s Disease Center and covered under the Human Tissue Act 2004.

## Author contributions

SJB conducted and coordinated the study, analyzed data, and contributed to writing the paper. JMB conducted the electrophysiology study and contributed to the behavior study. AJB contributed to the experimental design and generated the anti-S228 antibody. HES conducted the [S^35^]-GTPγS experiments on prion and AD samples. AJM conducted the AD radioligand binding experiments. DJW, NV, JAM, CM, and GRM supported prion mouse breeding, inoculation, and analysis of data. RP provided equipment for preliminary experiments. TMH provided technical support. JME and DJR supported histology and imaging. JW provided muscarinic knockout mice. PMS and AC contributed analysis of PAM data, provided BQZ-12, and contributed to writing the paper. LMB and CCF supported experimental design and analysis of data. JRS conducted and analyzed electrophysiological experiments. ABT conceived the study, led the program, and wrote the paper.

## Supplementary Material

Supplemental data

## References

[1] Altenbach C, Kusnetzow AK, Ernst OP, Hofmann KP, Hubbell WL (2008). High-resolution distance mapping in rhodopsin reveals the pattern of helix movement due to activation. Proc Natl Acad Sci U S A.

[2] Massoud F, Gauthier S (2010). Update on the pharmacological treatment of Alzheimer’s disease. Curr Neuropharmacol.

[3] Coyle JT, Price DL, DeLong MR (1983). Alzheimer’s disease: a disorder of cortical cholinergic innervation. Science.

[4] LaFerla FM, Green KN, Oddo S (2007). Intracellular amyloid-beta in Alzheimer’s disease. Nat Rev Neurosci.

[5] Nathan PJ (2013). The potent M1 receptor allosteric agonist GSK1034702 improves episodic memory in humans in the nicotine abstinence model of cognitive dysfunction. Int J Neuropsychopharmacol.

[6] van der Zee EA, Luiten PG (1999). Muscarinic acetylcholine receptors in the hippocampus, neocortex and amygdala: a review of immunocytochemical localization in relation to learning and memory. Prog Neurobiol.

[7] Wess J, Eglen RM, Gautam D (2007). Muscarinic acetylcholine receptors: mutant mice provide new insights for drug development. Nat Rev Drug Discov.

[8] Young SL, Bohenek DL, Fanselow MS (1995). Scopolamine impairs acquisition and facilitates consolidation of fear conditioning: differential effects for tone vs context conditioning. Neurobiol Learn Mem.

[9] Kruse AC (2012). Structure and dynamics of the M3 muscarinic acetylcholine receptor. Nature.

[10] Haga K (2012). Structure of the human M2 muscarinic acetylcholine receptor bound to an antagonist. Nature.

[11] Kruse AC (2013). Muscarinic receptors as model targets and antitargets for structure-based ligand discovery. Mol Pharmacol.

[12] Bodick NC (1997). Effects of xanomeline, a selective muscarinic receptor agonist, on cognitive function and behavioral symptoms in Alzheimer disease. Arch Neurol.

[13] Shekhar A (2008). Selective muscarinic receptor agonist xanomeline as a novel treatment approach for schizophrenia. Am J Psychiatry.

[14] Langmead CJ, Watson J, Reavill C (2008). Muscarinic acetylcholine receptors as CNS drug targets. Pharmacol Ther.

[15] Wood MD (1999). Functional comparison of muscarinic partial agonists at muscarinic receptor subtypes hM1, hM2, hM3, hM4 and hM5 using microphysiometry. Br J Pharmacol.

[16] May LT, Avlani VA, Sexton PM, Christopoulos A (2004). Allosteric modulation of G protein-coupled receptors. Curr Pharm Des.

[17] May LT, Leach K, Sexton PM, Christopoulos A (2007). Allosteric modulation of G protein-coupled receptors. Annu Rev Pharmacol Toxicol.

[18] Lange HS, Cannon CE, Drott JT, Kuduk SD, Uslaner JM (2015). The M1 muscarinic positive allosteric modulator PQCA improves performance on translatable tests of memory and attention in rhesus monkeys. J Pharmacol Exp Ther.

[19] Ma L (2009). Selective activation of the M1 muscarinic acetylcholine receptor achieved by allosteric potentiation. Proc Natl Acad Sci U S A.

[20] Puri V, Wang X, Vardigan JD, Kuduk SD, Uslaner JM (2015). The selective positive allosteric M1 muscarinic receptor modulator PQCA attenuates learning and memory deficits in the Tg2576 Alzheimer’s disease mouse model. Behav Brain Res.

[21] Vardigan JD (2015). Improved cognition without adverse effects: novel M1 muscarinic potentiator compares favorably to donepezil and xanomeline in rhesus monkey. Psychopharmacology (Berl).

[22] Wootten D, Christopoulos A, Sexton PM (2013). Emerging paradigms in GPCR allostery: implications for drug discovery. Nat Rev Drug Discov.

[23] Laurijssens B, Aujard F, Rahman A (2013). Animal models of Alzheimer’s disease and drug development. Drug Discov Today Technol.

[24] Mallucci GR (2002). Post-natal knockout of prion protein alters hippocampal CA1 properties, but does not result in neurodegeneration. EMBO J.

[25] Radford H, Moreno JA, Verity N, Halliday M, Mallucci GR (2015). PERK inhibition prevents tau-mediated neurodegeneration in a mouse model of frontotemporal dementia. Acta Neuropathol.

[26] Mallucci G, Dickinson A, Linehan J, Klöhn PC, Brandner S, Collinge J (2003). Depleting neuronal PrP in prion infection prevents disease and reverses spongiosis. Science.

[27] Halliez S (2013). Targeted knock-down of cellular prion protein expression in myelinating Schwann cells does not alter mouse prion pathogenesis. J Gen Virol.

[28] Bymaster FP (1994). Neurochemical effects of the M1 muscarinic agonist xanomeline (LY246708/NNC11-0232). J Pharmacol Exp Ther.

[29] Shannon HE (1994). Xanomeline: a novel muscarinic receptor agonist with functional selectivity for M1 receptors. J Pharmacol Exp Ther.

[30] Abdul-Ridha A, Lane JR, Sexton PM, Canals M, Christopoulos A (2013). Allosteric modulation of a chemogenetically modified G protein-coupled receptor. Mol Pharmacol.

[31] Canals M, Lane JR, Wen A, Scammells PJ, Sexton PM, Christopoulos A (2012). A Monod-Wyman-Changeux mechanism can explain G protein-coupled receptor (GPCR) allosteric modulation. J Biol Chem.

[32] Mistry SN, Valant C, Sexton PM, Capuano B, Christopoulos A, Scammells PJ (2013). Synthesis and pharmacological profiling of analogues of benzyl quinolone carboxylic acid (BQCA) as allosteric modulators of the M1 muscarinic receptor. J Med Chem.

[33] Abdul-Ridha A (2014). Mechanistic insights into allosteric structure-function relationships at the M1 muscarinic acetylcholine receptor. J Biol Chem.

[34] Phillips RG, LeDoux JE (1992). Differential contribution of amygdala and hippocampus to cued and contextual fear conditioning. Behav Neurosci.

[35] Butcher AJ (2016). An antibody biosensor establishes the activation of the M1 muscarinic acetylcholine receptor during learning and memory. J Biol Chem.

[36] Halliday M, Mallucci GR (2015). Review: Modulating the unfolded protein response to prevent neurodegeneration and enhance memory. Neuropathol Appl Neurobiol.

[37] Halliday M (2015). Partial restoration of protein synthesis rates by the small molecule ISRIB prevents neurodegeneration without pancreatic toxicity. Cell Death Dis.

[38] Radford HE, Mallucci GR (2010). The role of GPI-anchored PrP C in mediating the neurotoxic effect of scrapie prions in neurons. Curr Issues Mol Biol.

[39] Collinge J (2001). Prion diseases of humans and animals: their causes and molecular basis. Annu Rev Neurosci.

[40] Levey AI, Edmunds SM, Koliatsos V, Wiley RG, Heilman CJ (1995). Expression of m1-m4 muscarinic acetylcholine receptor proteins in rat hippocampus and regulation by cholinergic innervation. J Neurosci.

[41] Bartus RT, Dean RL, Beer B, Lippa AS (1982). The cholinergic hypothesis of geriatric memory dysfunction. Science.

[42] Mash DC, Flynn DD, Potter LT (1985). Loss of M2 muscarine receptors in the cerebral cortex in Alzheimer’s disease and experimental cholinergic denervation. Science.

[43] Andersen MB (2003). The muscarinic M1/M4 receptor agonist xanomeline exhibits antipsychotic-like activity in Cebus apella monkeys. Neuropsychopharmacology.

[44] Shannon HE (2000). Xanomeline, an M(1)/M(4) preferring muscarinic cholinergic receptor agonist, produces antipsychotic-like activity in rats and mice. Schizophr Res.

[45] Mirza NR, Peters D, Sparks RG (2003). Xanomeline and the antipsychotic potential of muscarinic receptor subtype selective agonists. CNS Drug Rev.

[46] Deacon RM (2006). Burrowing in rodents: a sensitive method for detecting behavioral dysfunction. Nat Protoc.

[47] Deacon RM, Croucher A, Rawlins JN (2002). Hippocampal cytotoxic lesion effects on species-typical behaviours in mice. Behav Brain Res.

[48] Riedel G, Platt B, Micheau J (2003). Glutamate receptor function in learning and memory. Behav Brain Res.

[49] Chung HJ, Steinberg JP, Huganir RL, Linden DJ (2003). Requirement of AMPA receptor GluR2 phosphorylation for cerebellar long-term depression. Science.

[50] Seidenman KJ, Steinberg JP, Huganir R, Malinow R (2003). Glutamate receptor subunit 2 Serine 880 phosphorylation modulates synaptic transmission and mediates plasticity in CA1 pyramidal cells. J Neurosci.

[51] Daw MI (2000). PDZ proteins interacting with C-terminal GluR2/3 are involved in a PKC-dependent regulation of AMPA receptors at hippocampal synapses. Neuron.

[52] Kim CH, Chung HJ, Lee HK, Huganir RL (2001). Interaction of the AMPA receptor subunit GluR2/3 with PDZ domains regulates hippocampal long-term depression. Proc Natl Acad Sci USA.

[53] Duprat F, Daw M, Lim W, Collingridge G, Isaac J (2003). GluR2 protein-protein interactions and the regulation of AMPA receptors during synaptic plasticity. Philos Trans R Soc Lond, B, Biol Sci.

[54] Conn PJ, Christopoulos A, Lindsley CW (2009). Allosteric modulators of GPCRs: a novel approach for the treatment of CNS disorders. Nat Rev Drug Discov.

[55] Chater TE, Goda Y (2014). The role of AMPA receptors in postsynaptic mechanisms of synaptic plasticity. Front Cell Neurosci.

[56] Abdul-Ridha A (2014). Molecular determinants of allosteric modulation at the M1 muscarinic acetylcholine receptor. J Biol Chem.

[57] Witkin KM, et al. In vitro pharmacological and rat pharmacokinetic characterization of LY3020371, a potent and selective mGlu2/3 receptor antagonist [published online ahead of print December 31, 2015]. *Neuropharmacology*. https://doi.org/10.1016/j.neuropharm.2015.12.021 10.1016/j.neuropharm.2015.12.021 26748052

