# Supplementary material for: M1 muscarinic allosteric modulators slow prion neurodegeneration and restore memory loss
Source: J Clin Invest. 2016 Dec 19;127(2):487–99. doi: 10.1172/JCI87526 (PMC5272187; doi:10.1172/JCI87526)
Supplement: Supplemental data [file jci-127-87526-s001.pdf]

Supplementary Information

Materials and Methods

Mouse Maintenance and Diet

Mice were fed ad libitum with a standard mouse chow. All animal work conformed to the United Kingdom Home Office regulations. The mAChR knockout mice were backcrossed for at least ten generations onto the black C57Bl6/NTAC background and were provided by Prof. Jurgen Wess. The Tg37 mouse line that over-expresses mouse prion protein has been described previously (1), and was provided by Prof. Giovanna Mallucci.

Human tissue sample information (Oregon Alzheimer's Disease Center)

Sample	Group	n	Age	PMI (h)	Amyloid Plaques	Neocortical tangles	Braak Stage
**Frontal cortex**	Control	10	54.9 [4.6]	25.8 (3.0)	0.2 (0.1)	0.1 (0.1)	
**Frontal cortex**	AD	10	72.4 [3.5]	25.9 (5.2)	2.6 (0.2)	5.9 (0.1)	5/6

Drug administration and pharmacokinetics

Compounds were administered via intraperitoneal (i.p.) injection 30 minutes prior to tissue/blood collection. Following this, mice were anaesthetised with 3% isofluorane (2L/min O_2), and blood was collected by cardiac puncture of the left ventricle. Blood was immediately transferred to EDTA tubes, centrifuged at 1000 xg for 10 minutes at 4°C, and supernatant was collected and frozen. From each mouse, brains were also dissected and snap frozen on dry ice.

Brain samples were homogenised in 3 volumes of methanol/water (1:4, v/v) by weight. A 25 µl aliquot of each study sample, calibration standard and

control sample were added to a 96-well plate then mixed with 180 µl of acetonitrile/methanol (1:1, v/v) containing internal standard. After mixing, the samples were centrifuged and the resulting supernatants were diluted 12.5-fold with methanol/water (1:1, v/v) prior to analyzing 10 µl aliquots by LC-MS/MS.

The sample extracts were analysed with an Applied Biosystems/MDS Sciex API 4000 triple quadrupole mass spectrometer. The analytes were chromatographically separated using a ThermoHypersil Betasil C18 2x20 mm 5 micron Javelin HPLC column, with a gradient LC system composed of water/trifluoroacetic acid/1 M ammonium bicarbonate, (1000:4:1, v/v) (Mobile Phase A), and acetonitrile/trifluoroacetic acid/1 M ammonium bicarbonate, (1000:4:1, v/v) (Mobile Phase B). Data were acquired and processed with Applied Biosystems/MDS Sciex Analyst software (version 1.4.2).

The unbound fraction of drug in brain was estimated using fast gradient elution LC-MS/MS to estimate the percent of compound bound to brain over a 4.5 hour incubation period at 37ºC, while undergoing orbital shaking. The assay was performed using a HT dialysis micro equilibrium device, using dialysis membrane strips (molecular weight cutoff 12 – 14k). At time 0 sample of brain homogenate was taken and samples taken from both the protein side and buffer side of the membrane after 4.5 hours of incubation. Drug concentrations were measured as described previously. Fraction unbound was calculated by dividing the concentration of the buffer side by the concentration of the protein side.

Prion infection of mice

Tg37 hemizygous mice were inoculated by intracerebral injection into the right parietal lobe with 1% brain homogenate of Rocky Mountain Laboratory (RML) prions aged 3–4 weeks as described previously (1). Control mice received 1% normal brain homogenate (NBH).

Fear conditioning learning and memory test

For behavioural testing of C57Bl6/NTAC or M_1-KO mice, 8 to 12 week-old male mice were used. For prion-infected mice, and the relevant control mice, behavioural experiments were conducted on male mice between 9 and 10 weeks post inoculation (w.p.i.) with prion infected or normal brain homogenate. Mice were acclimatised to the behavioural room for at least two hours prior to the test. For fear conditioning, mice were placed in the conditioning chamber (Stoelting ANY-maze fear conditioning system) and after a 2 minute adaptation period, received three tone/foot shock pairings where the foot shock (unconditioned stimulus; US; 2 seconds; 0.4 mA) always co-terminated with a tone (conditioned stimulus; CS; 2.8 kH; 85 dB; 30 seconds). The CS-US pairings were separated by 1 minute intervals. After completion of training, the mice remained in the conditioning chamber for 1 minute and were then returned to their home cages. The next day, mice were placed back in the conditioning chamber, and time spent immobile was recorded for 3 minutes to assess context-dependent learning. Data were analysed using ANY-maze software.

Pain threshold

The mice were placed on the grid floor of the conditioning chamber (described above for fear conditioning) and were given 2 second foot shocks of increasing intensity (0.10–0.4 mA) at 10 second intervals. The level of the electric current needed to elicit startle, running/jumping and vocalisation responses was determined. All animals were foot shock-naive before the experiment and were not used for any subsequent tests.

Burrowing

Assessment of burrowing was conducted on mice from 7 w.p.i. The burrowing test involved mice being placed into individual cages with a plastic cylinder filled with 140 g of food pellets. Food remaining in the cylinders after 2 hours was weighed and the amount displaced ('burrowed') was calculated. Prior to the burrowing test mice were placed in the burrowing cage for a 2 hour period. On the following day mice received vehicle or xanomeline (5 mg/kg) via i.p. injection 30 minutes prior to the burrowing test. This was then repeated on a weekly basis.

Open field

This test was used to analyse general locomotor activity levels. The mice were placed into a clear, Perspex square arena (50 x 50 cm) and activity was tracked for a 10 minute period using ANY-maze software.

Elevated plus maze

The elevated plus maze apparatus consisted of four non-transparent arms (50 x 10 cm); two enclosed arms (with black walls of 30 cm height) that formed a cross shape with two open arms opposite each other. The open arms were dimly illuminated. Mice were placed at the centre of the maze facing an open arm. Mice were tracked for 5 minutes, and their tendency towards dark, enclosed spaces, versus the open spaces, was used as a measure of anxiety. The number of entries of the animal from the central platform into the enclosed or open arms was counted, and data was recorded using the ANY-maze software.

Generation M_1 mAChR phospho-serine 228 specific antiserum

Phosphorylation specific antibody that detected phosphorylation of the M_1 mAChR at serine 228 was raised against the peptide AALQGS$_{(P)}$ETPGKG corresponding to amino acid residues 223-234 of the mouse M_1 mAChR. The 87 day program which included 4 immunisations was performed by Eurogentec. The resulting antiserum was purified against the immunising peptide.

Generation of M_1 mAChR antiserum

To generate antibodies for immunoprecipitation and detection of M_1 AChR protein, rabbits and rats were immunised with the peptide RDRGGKGQKPRGKEQ that corresponds to amino acids 334–348 of the

mouse M_1 mAChR. The resulting antiserum was purified against the immunising peptide.

Membrane preparation

All of the following procedures were performed at 4°C. For prion mouse hippocampus preparations (NBH, prion 9wpi or 10wpi) 8-10 mice were pooled. For human control or AD frontal cortex ~1.5g of tissue from each patient was used (Oregon Alzheimer's Disease Center). Frozen tissue was homogenised in ice-cold HEPES buffer (10 mM HEPES, 1 mM EGTA, 1 mM dithiothreitol) plus 10% (w/v) sucrose and 1 x protease inhibitor cocktail by 20 strokes with a hand held glass/Teflon tissue grinder. The homogenate was centrifuged at 1000 xg for 10 minutes and the supernatant collected. The resulting pellet was rehomogenised and centrifuged again at 1000 xg for 10 minutes. The combined supernatant was centrifuged at 11,000 xg for 20 minutes and the resulting pellet resuspended in final storage buffer (HEPEs buffer 2 plus 1 mM $MgCl_2$) before a final centrifugation at 27,000 xg for 20 minutes. The final pellet was resuspended in final storage buffer before performing protein estimation by the Bradford method using the BioRad Protein assay kit. The homogenate was then further diluted in final storage buffer to produce a concentration of 3 mg/ml.

Radioligand binding assays

Saturation analysis: membrane preparations of mouse hippocampus or cortex (50 µg/tube) were incubated in HEPEs buffer 1 (50 mM HEPEs, 110 mM NaCl, 5.4 mM KCl, 1.8 mM $CaCl_2$, 1 mM $MgSO_4$, 25 mM glucose, 58 mM

sucrose, pH 7.4) containing increasing concentrations (0.1-5 nM) of the muscarinic receptor antagonist [^{3}H]-N-methyl scopolamine ([^{3}H]-NMS) for 1 hour at 37°C. Membrane-bound ligand was separated from free ligand by rapid filtration onto GF/B glass microfiber filters followed by three rapid washes with ice-cold 0.9% NaCl. Membrane bound radioactivity was determined by liquid scintillation (Perkin Elmer Ultima Gold) counting. Nonspecific binding was determined by the inclusion of atropine (1 μM) during the incubation with [^{3}H]-NMS.

Competition experiments: hippocampal membranes were incubated with ~0.3 nM [^{3}H]-NMS and increasing concentrations of allosteric modulators, BQCA or BQZ-12 for 1 hour at 37°C. Non-specific binding and filtration was carried out as above.

For [^{3}H]-NMS binding to human tissue, experiments were performed in assay buffer of the following composition, 20 mM HEPES, 100 mM NaCl, 10 mM $MgCl_2$, pH 7.5 and used 10 μg/well of human protein in a total assay volume of 1000 μl. [^{3}H]-NMS (81.5 Ci/mmol; Perkin-Elmer, Boston, MA) was added to a final saturating concentration of 5 nM. Non-specific binding was defined in the presence of 10 μM atropine. Incubation was carried out for 2 hours at room temperature in quadruplicate. Binding reactions were terminated by rapid filtration through Filtermat B filters (PerkinElmer, Boston, MA) which were pre-soaked with 0.5% w/v PEI for 1 h. Filters were then washed 3 times with 1 ml ice-cold assay buffer. Dried filters were counted with Meltilex B scintillant using a Trilux 1450 scintillation counter (PerkinElmer, Boston, MA). The specific bound counts (dpm) were expressed as fmol/mg protein. Data analysis was accomplished using Excel and GraphPad Prism 6.

[^{35}S]-GTPγS assay

Concentration dependent effects of agonists and PAMs at the M_1 mAChR were measured by [^{35}S]-GTPγS binding to $G\alpha_q$ proteins using the SPA antibody capture method in 96-well white clear bottom plates (DeLapp *et al.*, 1999). Briefly, mouse (hippocampi) or human (frontal cortex) membranes were thawed in a water bath at 37°C and then diluted in assay buffer (20 mM HEPEs, 100 mM NaCl, 5 mM $MgCl_2$, pH 7.4) to 100 mg/ml. The diluted membrane was then homogenized briefly (10 strokes with a hand held glass/Teflon tissue grinder) before incubation with acetylcholine esterase (0.1 U/ml) for 30 minutes at room temperature. After the incubation, 100 µl/well of membrane (10 µg) was added to the assay plate followed by 50 µl agonist/PAM at the appropriate concentration and 50 µl of [^{35}S]-GTPγS (0.5 nM final concentration). The plate was incubated for 60 minutes at room temperature before addition of the SPA bead/antibody/NP40 mix (Anti rabbit SPA PVT beads 1.25 mg/well, $G\alpha_q$ antibody (Santa Cruz) 1 mg/ml final and Nonidet P40 0.27% final concentration). The plate was then mixed and incubated for a further 3 hours at room temperature. Plates were then centrifuged at 200 xg for 10 minutes at room temperature before reading on a Trilux scintillation counter (Wallac 1450 microbeta counter, Perkin Elmer, 1 minute counts). Data was converted to % response compared to Oxotremorine M 100 mM or % over basal in Microsoft Excel before being transferred to Graphpad Prism software to generate an EC_{50} (4 parameter curve fit, sigmoidal dose response, variable slope).

[^{35}S]-GTPγS assay (for supplementary Figure 5 only)

Hippocampi from C57Bl/6NTAC or M_1-KO mice (8-12) were suspended in ice-cold harvesting buffer (containing 0.9% (w/v) NaCl, 10 mM HEPES, 0.2% (w/v) EDTA, pH 7.4) and homogenized (4 x 5 sec bursts) using a Polytron homogeniser. The suspension was centrifuged at 200 xg for 5 min at 4°C using an Eppendorf 5810R bench-top centrifuge. Supernatants were collected and re-homogenised as above. The suspension was subsequently centrifuged for 20 min at 40,000 xg at 4°C using a Beckman Coulter Avanti JXN-26 centrifuge with a JA-25.25 rotor. Supernatant was discarded, and the pellet was re-suspended in 10 mL ice-cold buffer (10 mM HEPES, 10 mM EDTA, pH 7.4). The pellet was homogenised, GTP (1 mM final) was added and the suspension was incubated at 37°C for 15 min. The suspension was subsequently centrifuged for 20 min at 40,000 xg at 4°C and the pellet was re-suspended in 15 mL ice-cold re-suspension buffer (10 mM HEPES, 0.1 mM EDTA, pH 7.4) and re-homogenised as before. Suspension was centrifuged again for 20 min at 40,000 xg at 4°C. The final pellet was resuspended in re-suspension buffer and protein concentration was estimated using a Bradford assay. The homogenate was then further diluted in final storage buffer to produce a concentration of 2 mg/ml.

[^{35}S]-GTPγS binding and immunoprecipitation of Gα subunits was performed as previously described. Specifically, WT or M_1-KO membranes were diluted in assay buffer (10 mM HEPES, 100 mM NaCl, 10 mM $MgC1_2$, pH 7.4) containing a final concentration of 1 μM GDP. Membranes (75 μg in a total assay volume of 200 μL) were added to [^{35}S]-GTPγS (1 nM final concentration) and agonists and incubated at 30°C for 5 min. Reactions were terminated by the addition of 1 mL ice-cold assay buffer and immediate

transfer to an ice bath. Samples were centrifuged (20,000 xg, 6 min, 4°C) and membrane pellets solubilised by the addition of 50 µL ice-cold solubilisation buffer (100 mM Tris HCl, 200 mM NaCl, 1 mM EDTA, 1.25% Igepal and 0.2% SDS, pH 7.4) and incubation for 1 h at 4°C on a shaking platform. Following complete protein re-solubilisation, 50 µL of solubilisation buffer without SDS was added. Solubilised protein was pre-cleared using normal rabbit serum at a dilution of 1:100 and 3% (w/v) protein A-sepharose beads in TE buffer (10 mM Tris HCl, 10 mM EDTA, pH 8.0) added for 60 min at 4°C. Protein A-sepharose beads and insoluble material were collected by centrifugation (20,000 xg, 6 min, 4°C) and 100 µL of the supernatant was transferred to fresh tubes containing G_q-specific anti-serum (Santa Cruz; sc393) and incubated overnight at 4°C. Protein A-sepharose beads were added to samples, vortex mixed and rotated at 4°C for 90 min before being centrifuged (10,000 xg, 1 min, 4°C). Supernatants were aspirated and the protein A-sepharose beads washed three times with ice-cold solubilisation buffer (without SDS). Recovered beads were then mixed with 1 mL FloScint-IV scintillation cocktail and counted by liquid scintillation spectrometry.

Electrophysiology

Brain slices were prepared from animals killed by decapitation (age < P20) or by cervical dislocation. Whole-cell patch recordings were made from visually identified mouse CA1 neurons in acute brain slices (250 µm thick) of the hippocampus (2-3 cells were measured per mouse in a total of 7 animals) essentially as described previously (2). Experiments were performed at 32 ± 1°C (LinLab software, Scientifica). Synaptic stimulation (using a DS2A

isolated stimulator (Digitimer, Welwyn Garden City, UK; 1–10 V, 0.1–0.2 ms) was delivered via a bipolar platinum electrode placed at the Schaffer collateral and cells were voltage clamped at -60 mV. Patch pipettes were pulled from glass capillaries (GC150F-7.5, o.d. 1.5 mm, Harvard Apparatus, Edenbridge, UK) and had resistances of 3.5–5 MΩ when filled with the pipette solution. Series resistances were between 15 and 20 MΩ (series resistance compensation and prediction were around 70%). Data were recorded using a Multiclamp 700B amplifier (Molecular Devices, Sunnyvale, CA, USA). Stimulation, data acquisition and analysis were performed using pCLAMP 10.4 and Clampfit 10.4 (Molecular Devices). Average data are presented as mean ± S.E.M.

An artificial cerebrospinal fluid (aCSF) was used for slice incubation, maintenance after slicing and perfusion during recordings (125 mM NaCl, 2.5 mM KCl, 26 mM $NaHCO_3$, 10 mM glucose, 1.25 mM NaH_2PO_4, 2 mM sodium pyruvate, 3 mM *myo*-inositol, 2 mM $CaCl_2$, 1 mM $MgCl_2$, 0.5 mM ascorbic acid, pH 7.4 when gassed with 95% O_2, 5% CO_2. Osmolarity was around 310 mosmol l^{-1}. A *low-sodium aCSF* was used during preparation of slices, with a composition as above for aCSF, except that NaCl was replaced by 200 mM sucrose, and $CaCl_2$ and $MgCl_2$ were changed to 0.1 mM and 4 mM, respectively. The *pipette solution* for whole-cell recordings contained 120 mM potassium methanesulfonate, 10 mM HEPEs, 0.2 mM EGTA, 4 mM K-ATP, 0.3 mM Na-GTP, 8 mM NaCl, 10 mM KCl, pH 7.4 with osmolarity between to 280-290 mosmol l^{-1}. aCSF buffer was supplemented with D-AP5 (50 µM) and picrotoxin (50 µM) to block NMDA- and GABA-receptors, respectively. Xanomeline stock (1 mM) was prepared in 5% glucose and was diluted to 100

nM in aCSF. Following baseline recordings, muscarinic receptor ligands were perfused for at least 6 minutes before starting the paired recording. In this way the impact of mAChRs on AMPA currents in hippocampal CA1 pyramidal neurons was determined by calculating the area under the current curve (AUC, time-integral; reflecting synaptic strength also known as current density analysis) of the EPSC compared to control.

Immunohistochemistry

For drug administration, mice were injected (i.p.) with muscarinic receptor compounds 30 minutes prior to anaesthesia and perfusion fixation.

For fear conditioning, mice were placed into the fear conditioning chamber and underwent the training protocol as described above. Control mice were placed into the chamber and received an immediate foot shock (2 sec; 0.4 mA). Approximately 30 seconds after training or the immediate foot shock, mice were returned to their home cage.

Mice were anaesthetised with 3% isofluorane (2L/min O_2) and transcardially perfused with 20 ml of ice-cold PBS, followed by 20 ml of ice-cold 4% PFA. Following fixation, brains were immediately removed, and further fixed overnight in 4% PFA at 4°C. Brains were processed in paraffin wax and sliced at 5 μM using a microtome. Slices were either stained with haematoxylin and Eosin (H&E) or underwent antigen retrieval. Following antigen retrieval, sections were washed in TBS + 0.1% triton x-100 and blocked for 2 h at RT in TBS, 0.1% triton X-100, 10% goat serum and 5% BSA. Sections were incubated with antibodies to c-Fos (Santa Cruz; sc253), ARC (Santa Cruz; sc15325), (p)-Ser228 (in-house), ChAT (Millipore; AB143), NeuN (Millipore;

MAB377) or GFAP (Sigma; G3893) (1:100 in blocking buffer overnight at 4°C). Sections were washed three times, and incubated with Alexa Fluor fluorescent secondary antibodies for 1 h at RT in blocking buffer. Following three washes, slices were mounted in Vectorshield hardset mounting medium with DAPI. All images were taken using either a Zeiss Axiovert 200M microscope with a Colibri illumination system with Axiovision 4.8 software (Zeiss) or a Zeiss LSM 510 META NLO microscope with Zen 2009 software (Zeiss). Quantification of CA1 pyramidal neurons was determined by identification of NeuN-positive cells using Velocity software. All analyses were performed using hippocampi from three mice in triplicate.

Immunoblotting

Mice were culled via cervical dislocation, and hippocampi and cortex were dissected and snap frozen on dry ice. Protein samples were isolated from hippocampi or cortex using protein lysis buffer (50 mM Tris HCl, 1 mM EDTA, 1 mM EGTA, 0.1% β-mercaptoethanol, 1% triton X-100, pH 7.5) supplemented with Phos-STOP and protease inhibitors (Roche). Concentrations of proteins in the lysate were determined using a Bradford assay, and then normalised to 1μg/μl in sample buffer (62.5 mM Tris HCl, 1% SDS, 0.001% bromophenol blue, 10% glycerol, 2.5% β-mercaptoethanol, pH 6.8).

Samples were boiled for 3 minutes and centrifuged at 21,000 xg for 2 minutes. Protein levels were determined by resolving 5-20 mg of protein on SDS–polyacrylamide electrophoresis gels (8-12%) and transferred onto nitrocellulose membrane, using a Bio-Rad semi-dry transfer system.

Membranes were blocked in 5% milk/TBST or 5% BSA/TBST (for phosphorylation-specific antibodies) for a minimum of 1 hour at room temperature. Membranes were incubated overnight at 4°C with antibodies raised to the M_1 mAChR (in-house), AMPA receptor subunits (Santa Cruz; GluR1 (sc13152) or GluR2 (sc7611)), phosphorylation-sites of the AMPA receptor subunits (Millipore; (p)-Ser880 GluR2 (07-294), (p)-Ser831 GluR1 (04-823), (p)-Ser845 GluR1 (04-1073)), PrP (Abcam; ab61409) or α-tubulin (Santa Cruz; sc8035).

For assessing PrPsc levels, hippocampi and cortex were homogenised in lysis buffer (as above) and then treated with proteinase K (100 μg/ml) for 1 hour at 37°C. Samples were then resuspended into sample buffer and treated as above.

Supplementary Figures

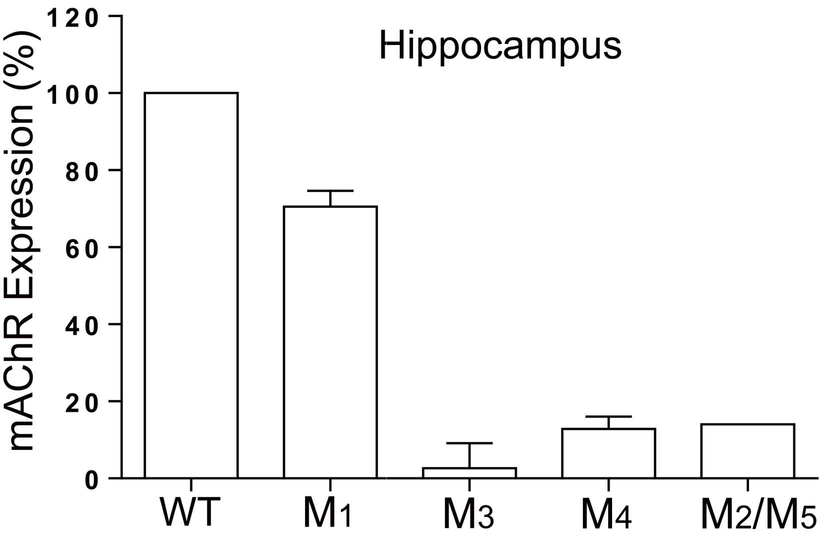

Supplementary Figure 1. Muscarinic acetylcholine receptor (mAChR) expression in membranes derived from the hippocampus of wild type (WT), M_1-, M_3-, M_4-KO mice were assessed using [^{3}H]-NMS binding. The level of receptor subtype expression was calculated by subtracting the level of residual [^{3}H]-NMS binding in the KO from that in the WT mice. The mAChR population remaining after taking into account M_1, M_3 and M_4 mAChRs was designated as M_2/M_5-KO. Data shown are the mean ± S.E.M. (n=3 mice per group).

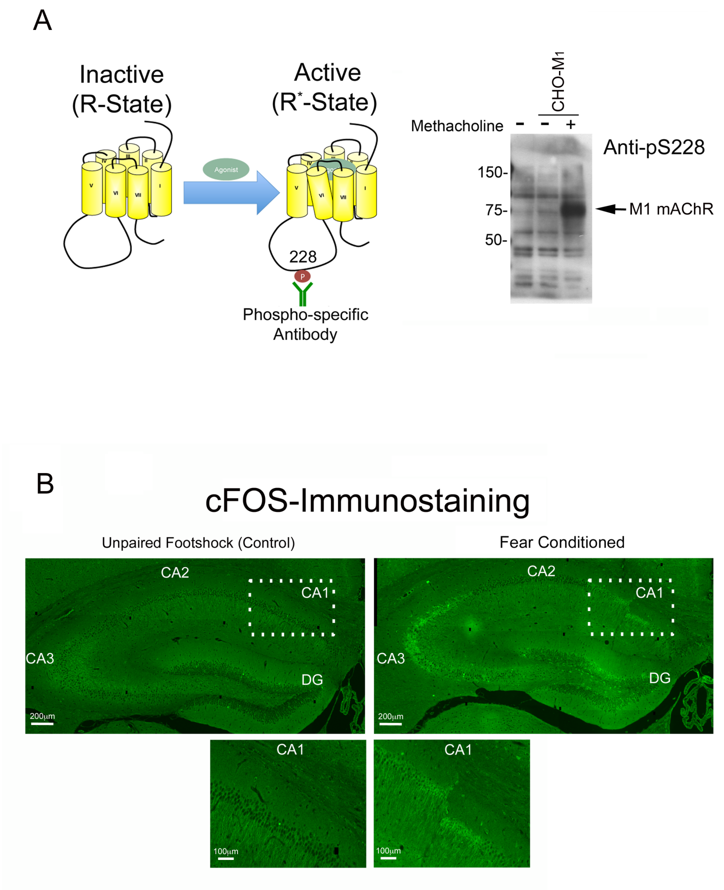

Supplementary Figure 2. A. An illustration of the antibody biosensor (anti-pS228) used to distinguish between the inactive, R-state, from the active, R*-state, of the M_1 mAChR. This biosensor was an antibody raised against phosphorylated serine 228 (S228) in the third intracellular loop of the M_1 mAChR – a phosphorylation event that occurs when the receptor is occupied by agonist. Western blot using the anti-pS228 antibody to probe lysates from CHO cells or from CHO cells expressing the M_1 mAChR (CHO-M_1) following stimulation with vehicle or the muscarinic agonist methacholine (100 μM, 5 minutes). **B.** Fear conditioning training increased neuronal activity, as assessed by an increase in cFOS immunostaining, in the same regions of the hippocampus as those observed for activated M_1 mAChR (see; Fig. 1E). Results shown are typical of at least 3 independent experiments.

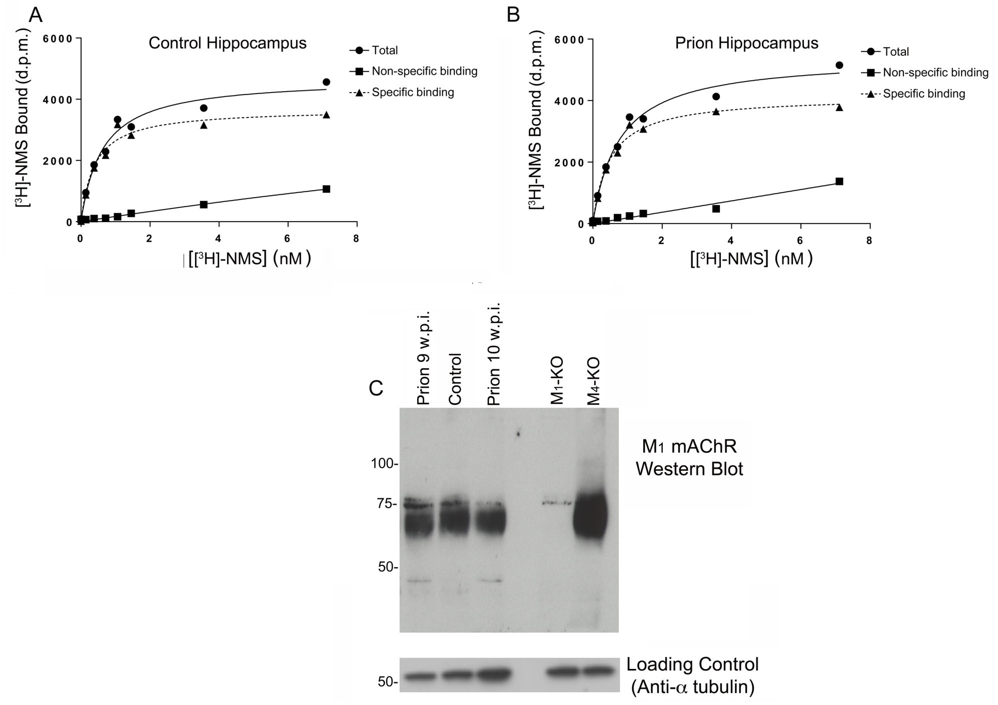

Supplementary Figure 3. A. Representative saturation binding experiment showing [^{3}H]-NMS binding to hippocampal membranes (20 μg protein) prepared from mice injected with normal brain homogenate (control). Non-specific binding was determined by the addition of atropine (1 μM). Data are the mean ± S.E.M (n=3). **B.** Same as (**A**) but membranes were prepared from prion-infected mice (10 w.p.i.). **C.** Lysates prepared from control mice or prion-infected mice (10 w.p.i.) were probed in Western blot analysis for the expression of M_1 mAChR using an M_1 mAChR specific antibody. Data shown is representative of at least three independent experiments.

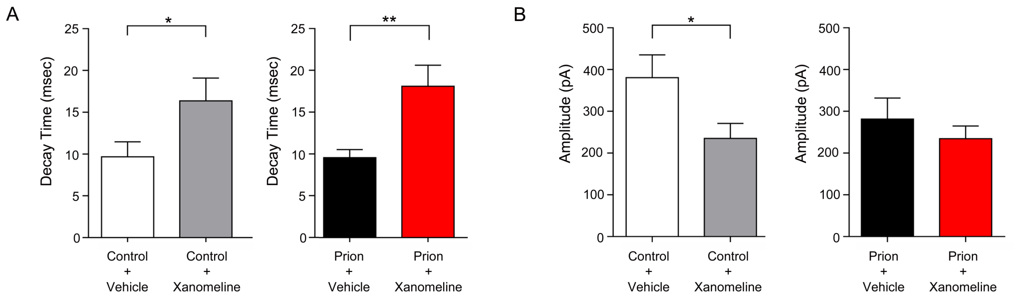

Supplementary Figure 4. A. Decay time of the AMPA-receptor mediated glutamatergic current was increased by xanomeline in hippocampal slices prepared from control (left) and prion-infected (right) mice. **B.** The amplitude of AMPA-receptor mediated glutamatergic currents was decreased significantly in control (left) but not in hippocampal slices from prion diseased (fight) mice (control n=10 cells; prion n=12 cells). Data are expressed as mean ± S.E.M. and statistical analysis used was paired Student's t test (*P<0.05, **P<0.01)

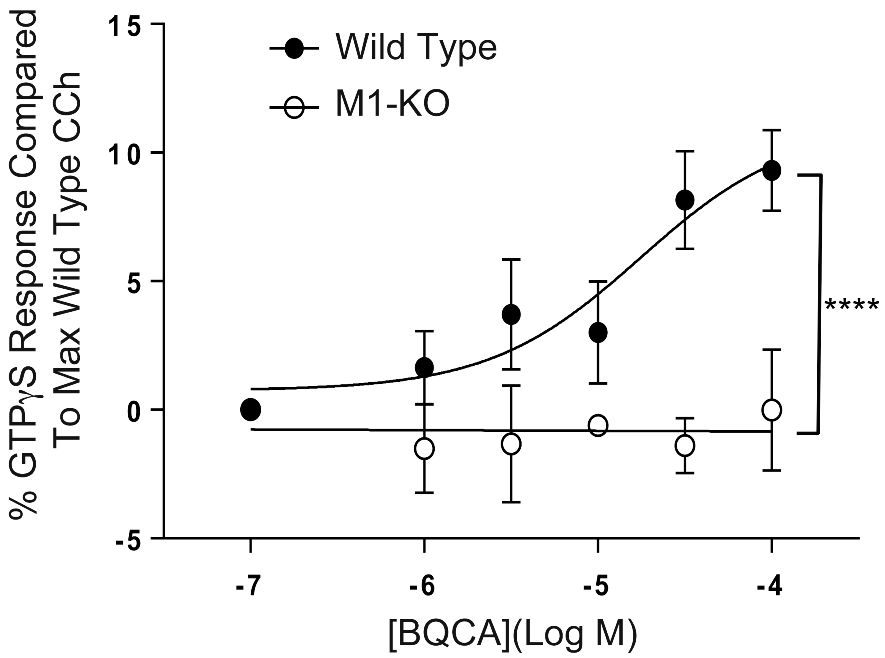

Supplementary Figure 5. Stimulation of [^{35}S]-GTPγS binding to membranes prepared from WT- or M_1-KO mice in response to BQCA are expressed as a percentage of the maximal response observed with the full agonist carbachol (CCh). The mean pEC_{50} value for BQCA-stimulation in wild-type membranes was 4.76 ± 0.42 (n=3). Data shown are means ± S.E.M. (n=3). Data was analysed using a two way ANOVA (*****P*<0.0001)

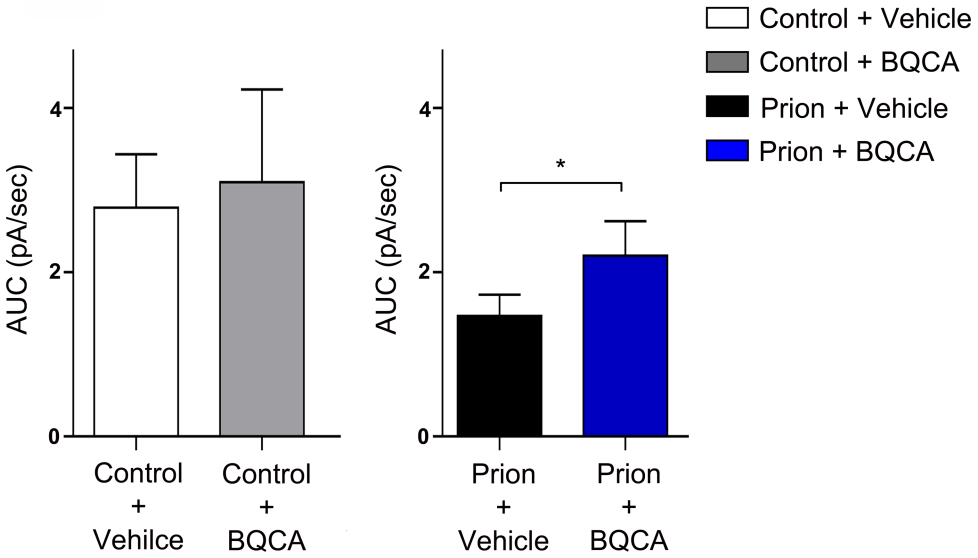

Supplementary Figure 6. Area under the current curve (AUC) of AMPA receptor-mediated currents before and after treatment with BQCA (control n=7, prion n=9). Statistical analysis by paired Student's t test; *P<0.05.

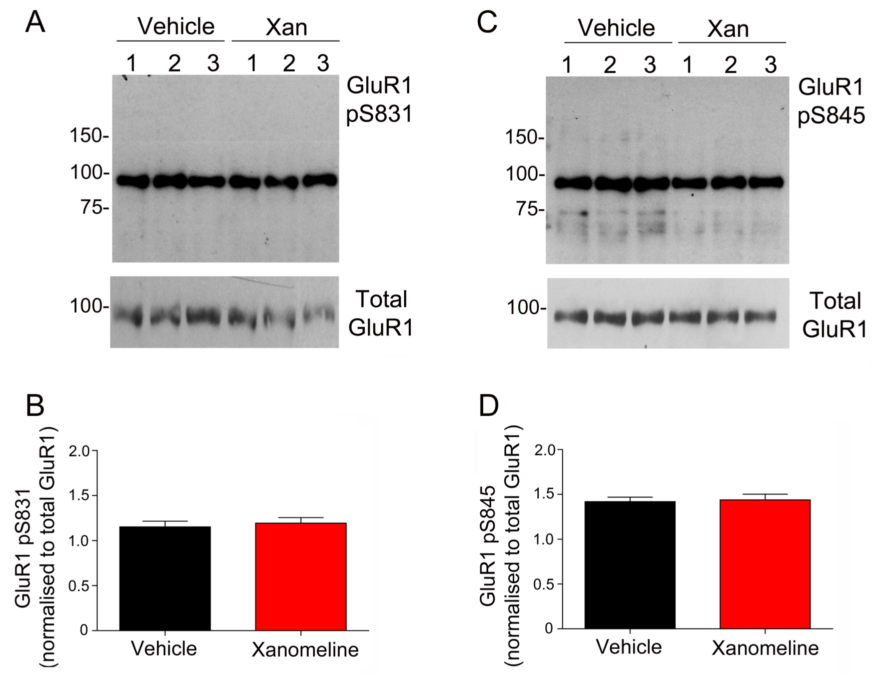

Supplementary Figure 7. A. Western blot of hippocampal lysates prepared from prion-infected mice treated with vehicle or xanomeline (Xan, 5 mg/kg) and probed with an antibody that detects phosphorylation of the GluR1 AMPA receptor subunit at serine 831. Blots were stripped and re-probed with anti-GluR1 antibodies as a loading control. **B.** Quantification of **(A)**. **C.** Same as **(A)** but blots were probed with an antibody that detects phosphorylation of the GluR1 AMPA receptor subunit at serine 845. **D**. Quantification of **(C)**. Data are expressed as the mean ± S.E.M. (n=3).

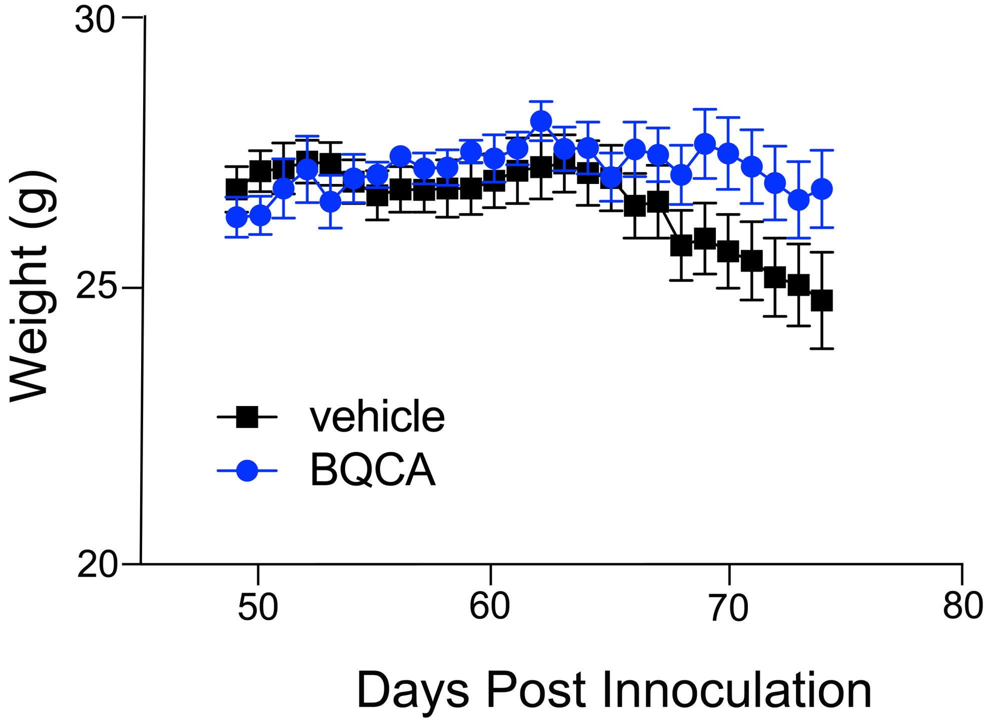

Supplementary Figure 8. Weight of prion infected mice from 7 w.p.i. (49 days) injected daily with either vehicle (black squares) or BQCA (blue circles) (15mg/kg, i.p.). Data are expressed as the mean ± S.E.M. (n=10).

Supplementary Table 1. Brain exposure levels of xanomeline and BQCA

The mean plasma, brain and free brain concentrations 30 minutes after i.p. injection of xanomeline (5 mg/kg) and BQCA (15 mg/kg) (n=3).

Compound	Mean Plasma (nM)	SD	%CV	Mean Brain (nM)	SD	Mean Unbound Brain (nM)	SD
Xanomeline	29.2	8.06	27.60	766	86.0	3.06	0.35
BQCA	2370	163	6.88	354	24.2	51.82	3.56

Supplementary Table 2.

Hippocampal membranes derived from control and prion infected mice and from AD patients were used to generate GTPγS concentration response curves (shown in Figures 5B, C). Here the mean pEC_{50} values (± S.E.M.; n=3) for agonists in the absence and presence of BQCA (3μM) are shown

	NBH	Prion 9 w.p.i.	Prion 10 w.p.i.
**OXO-M**	6.43 ± 0.045	6.62 ± 0.044	6.48 ± 0.048
**OXO-M + BQCA (3μM)**	8.02 ± 0.037	8.16 ± 0.038	8.06 ± 0.029

	Control	AD
**ACh**	5.94 ± 0.12	5.82 ± 0.17
**ACh + BQCA(3μM)**	7.79 ± 0.11	7.65 ± 0.08

Supplementary Table 3.

Multiple concentrations of donepezil, xanomeline, BQCA or BQZ-12 were administered via intraperitoneal injection (in 5% glucose) to wild-type mice and were observed for a range of side-effects. Shown are the proportion of mice displaying each side-effect following administration of drug (n=3-5).

Compound	Donepezil			Xanomeline				BQCA			BQZ-12	
Dose (mg/kg)	0.5	1.0	2.5	5.0	10.0	15.0	30.0	15.0	20.0	30.0	1.5	5.0
Piloerection	-	3/3	0/5	-	3/3	3/3	3/3	-	-	-	-	-
Squinting of eyes	-	3/3	5/5	-	3/3	3/3	3/3	-	-	-	-	-
Subdued	-	3/3	5/5	-	3/3	3/3	3/3	-	-	-	-	-
Hunched posture	-	3/3	5/5	-	3/3	3/3	3/3	-	-	-	-	-
Impaired mobility	-	3/3	5/5	-	0/3	0/3	0/3	-	-	-	-	-
Lifting head	-	1/3	0/5	-	0/3	0/3	0/3	-	-	-	-	-
Laboured respiration	-	0/3	5/5	-	0/3	0/3	0/3	-	-	-	-	-
Ataxia	-	0/3	5/5	-	0/3	0/3	0/3	-	-	-	-	-
Paralysis	-	0/3	5/5	-	0/3	0/3	0/3	-	-	-	-	-

- All compounds dosed via i.p. in 5% glucose.
- Adverse effects of 2.5mg/kg of donepezil were apparent within 5 minutes of dosing and were sufficiently adverse to initiate termination of the experiment.
- Side effects of xanomeline at a dose of 10mg/kg and above were apparent after approx. 20-30 minutes, but were unsustained. All mice had recovered by 60 minutes after administration.

References

1. **Mallucci, G., Dickinson, A., Linehan, J., Klohn, P.C., Brandner, S., and Collinge, J. 2003. Depleting neuronal PrP in prion infection prevents disease and reverses spongiosis. *Science* 302:871-874.**
2. **Steinert, J.R., Robinson, S.W., Tong, H., Haustein, M.D., Kopp-Scheinpflug, C., and Forsythe, I.D. 2011. Nitric oxide is an activity-dependent regulator of target neuron intrinsic excitability. *Neuron* 71:291-305.**
